# Unexpected Isomerization of Hexa‐*tert*‐butyl‐octaphosphane

**DOI:** 10.1002/chem.201904531

**Published:** 2019-12-27

**Authors:** Toni Grell, Evamarie Hey‐Hawkins

**Affiliations:** ^1^ Faculty of Chemistry and Mineralogy Institute of Inorganic Chemistry Leipzig University Johannisallee 29 04103 Leipzig Germany

**Keywords:** gold, isomerization, oligophosphanes, palladium, phosphorus

## Abstract

Octaphosphane {*cyclo*‐(P_4_ *t*Bu_3_)}_2_ (**1**) undergoes an unexpected isomerization reaction to the constitutional isomer 2,2′,2′′,2′′′,3,3′‐hexa‐*tert*‐butyl‐bicyclo[3.3.0]octaphosphane (**2**) in the presence of Lewis acidic metal salts. The mechanism of this reaction is discussed and elucidated with DFT calculations. The results underline the fascinating similarity between phosphorus‐rich and isolobal carbon compounds. The new bicyclic octaphosphane **2** shows a dynamic behavior in solution and reacts with [AuCl(tht)] (tht=tetrahydrothiophene) to give a mono‐ ([AuCl(**2**‐κ*P* ^3^)], **3**) and a dinuclear complex ([(AuCl)_2_(**2**‐κ*P* ^3^,κ*P* ^3′^)], **4**). With *cis*‐[PdCl_2_(cod)] (cod=1,5‐cyclooctadiene), the chelate complex ([PdCl_2_(**2**‐κ^2^ *P* ^2^,*P* ^2′^)], **5**) with a different coordination mode of the ligand was obtained.

## Graphical abstract

**The octaphosphane** {*cyclo*‐(P_4_ *t*Bu_3_)}_2_ unexpectedly isomerizes to a new constitutional isomer in the presence of Lewis acidic metal salts. The mechanism of this reaction was elucidated and furthermore compared with the isolobal organic compounds (see scheme). This new octaphosphane 2,2′,2′′,2′′′,3,3′‐hexa‐*tert*‐butyl‐bicyclo[3.3.0]octaphosphane can coordinate up to two gold(I) chloride fragments, whereas a tricyclic complex was formed with palladium(II).

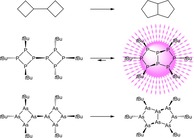

The chemistry of phosphorus‐rich compounds is an interesting field with a history of almost 150 years. Since the discovery of “Phosphabenzol”^1^ and the pioneering work of Baudler,^2^ who reported a vast number of these molecules, several groups focus on this topic today because of its outstanding importance in the direct activation of white phosphorus (P_4_)^3^ or the preparation of phosphorus‐rich metal phosphides.^4^, ^5^ Surely one of the most fascinating features of these compounds is their similarity to organic molecules as they can exhibit constitutional, configurational as well as conformational isomerism and also multiple bonds^6^ and even aromaticity.^7^ This analogy can be rationalized with the isolobal concept and is the reason why phosphorus is often referred to as the carbon copy.^8^

Herein, we report a discovery that further substantiates this statement. Recently, we reported the facile synthesis of hexa‐*tert*‐butyl‐octaphosphane (**1**), which reacted with [AuCl(tht)] (tht=tetrahydrothiophene) yielding mono‐, di‐, and trinuclear complexes, in which up to four phosphorus atoms of the ligand are involved in coordinating gold(I) atoms.^9^ During these studies, we observed a limited coordination behavior of **1**. Instead of coordination, the constitutional isomer octaphosphane **2** was formed upon reaction with a large variety of Lewis acidic metal salts and complexes, such as MX_2_ or [MX_2_(L)] (M=Mg, Mn, Fe, Co, Zn, Cd, Sn; X=Cl, Br; L=2,2′‐bipyridine or *N*,*N*,*N*′,*N*′‐tetramethylethane‐1,2‐diamine; Scheme 1).

**Scheme 1:**
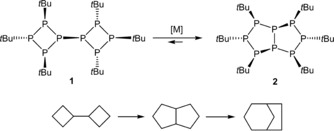
Isomerization reaction between octaphosphane **1** and **2**, as well as for the isolobal bi(cyclobutane) to bicyclo[3.3.0]octane (pentalane) and bicyclo[3.2.1]octane.

Isomerization reactions of phosphorus‐rich molecules are known in the literature.^4^, ^10^ One famous example is the Cope‐like rearrangement of the P_7_^3−^ anion.^11^ In addition to that, the heavier homologue of octaphosphane **1**, {*cyclo*‐(As_4_ *t*Bu_3_)}_2_ spontaneously isomerizes in solution at room temperature to the homologue of **2**.^12^ Furthermore, the isolobal octane, bi(cyclobutane), irreversibly isomerizes to bicyclo[3.3.0]octane (pentalane) when treated with the Lewis acid AlBr_3_ with formation of bicyclo[3.2.1]octane as the final product (Scheme 1).^13^

In the case of the octaphosphane **1**, the isomerization requires a Lewis acidic metal salt, which influences the reaction rate and temperature. The reaction of **1** with different equivalents of ZnCl_2_ (in C_6_D_6_, reflux) was monitored by ^31^P{^1^H} NMR spectroscopy. In each case, the conversion stopped at approximately 90 %, and no complete conversion of **1** to **2** was observed. The amount of the salt merely influenced the time until this state was reached (Figure S1, Supporting Information). Conversely, under the same conditions the conversion of octaphosphane **2** stops at 10 %. These observations thus imply an equilibrium between octaphosphanes **1** and **2** in the presence of a Lewis acidic metal salt. Accordingly, octaphosphane **2** was also obtained as a side product in the synthesis of **1**, which involves the two metal salts SnCl_2_ and MgCl_2_.^9^

In analogy to the mechanism that was postulated for bi(cyclobutane),^13^ we propose the mechanism depicted in Scheme 2 for the isomerization of **1** and **2**. Firstly, a monometallic complex (**i1**) is formed, in which the metal cation is coordinated by a bridge P atom and one of the *t*Bu carrying P atoms usually involved in coordination (β‐P).^9^ This leads to a proximity of the second bridge P atom to the other *t*Bu carrying P atom thus facilitating bond cleavage and formation to give the spirocyclic intermediate **i2**. In this intermediate, cleavage of the conceivable P−P bonds would either lead to a diastereomer with wrong configuration (bond I in Scheme 2) or is impaired, as the phosphanido group is sterically blocked by the metal cation (bond II in Scheme 2). Therefore, a rearrangement to intermediate **i3** is required followed by formation of intermediate **i4**. Liberation of the metal and inversion of the phosphorus atom P^#^ finally gives **2**.

**Scheme 2:**
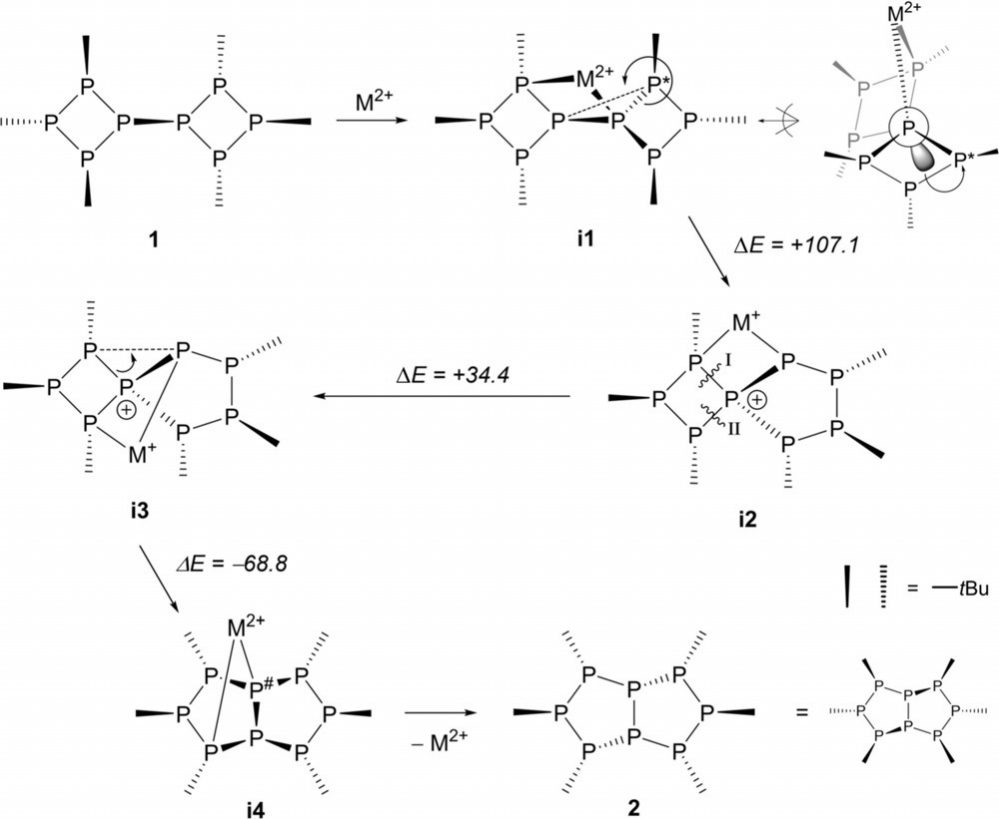
Proposed mechanism of the isomerization reaction between octaphosphanes **1** and **2**. Reaction energies (calculated with Zn^2+^ as M^2+^ and two Cl^−^ co‐ligands) are given in kJ mol^−1^. Bond formation (dashed line) and cleavage (wavy lines) are indicated.

DFT calculations involving a ZnCl_2_ fragment showed that the reaction energies (Scheme 2) for the mechanism are plausible, especially given the elevated temperatures required. Although the inversion barriers of phosphanes are usually high, they can be much smaller in oligophosphanes, and thus the inversion of P^#^ is conceivable.^14^

The analogues of octaphosphane **2** with Me, Et, and *i*Pr instead of *t*Bu substituents are known, but could not be characterized by X‐ray diffraction.^15^ The identity of **2** was unambiguously verified by single‐crystal XRD. The molecular structure (Figure S18, Supporting Information) confirms the proposed connectivity of the P_8_ skeleton in a *C*_2_‐symmetrical octaphosphane. All structural parameters including the P−P bond lengths between the bridgehead phosphorus atoms are in the expected range. The structure is furthermore analogous to As_8_ *t*Bu_6_ with respect to configuration and conformation. The ^31^P{^1^H} NMR spectrum of octaphosphane **2** shows three symmetrical multiplets, which could be successfully simulated as an AA′A′′A′′′BB′CC′ spin system assuming *C*_2*v*_ symmetry (Figure 1). The obtained parameters confirm the constitution, as well as the configuration of the oligophosphane, as the ^1^ *J*_PP_ coupling constants range between −262.67 and −311.99 Hz, which is in excellent agreement for similar compounds with *t*Bu substituents in a *trans* arrangement.^16^ Moreover, the ^2^ *J*_PP_ coupling constant (*J*_AA′′_) is large and positive indicating lone pairs of electrons directed towards each other,^17^ thus corroborating the *endo* configuration of the molecule. Finally, the values are also similar to the respective ethyl‐substituted oligophosphane.^18^ This is also the case for the chemical shifts. Although *δ*(P_A_) and *δ*(P_B_) are similar to the analogues, the signal P_C_ appears at higher field. This matches the trend for Me, Et, and *i*Pr, in which an increasing steric demand of the substituent causes a growing shielding of this signal.^15^

**Figure 1:**
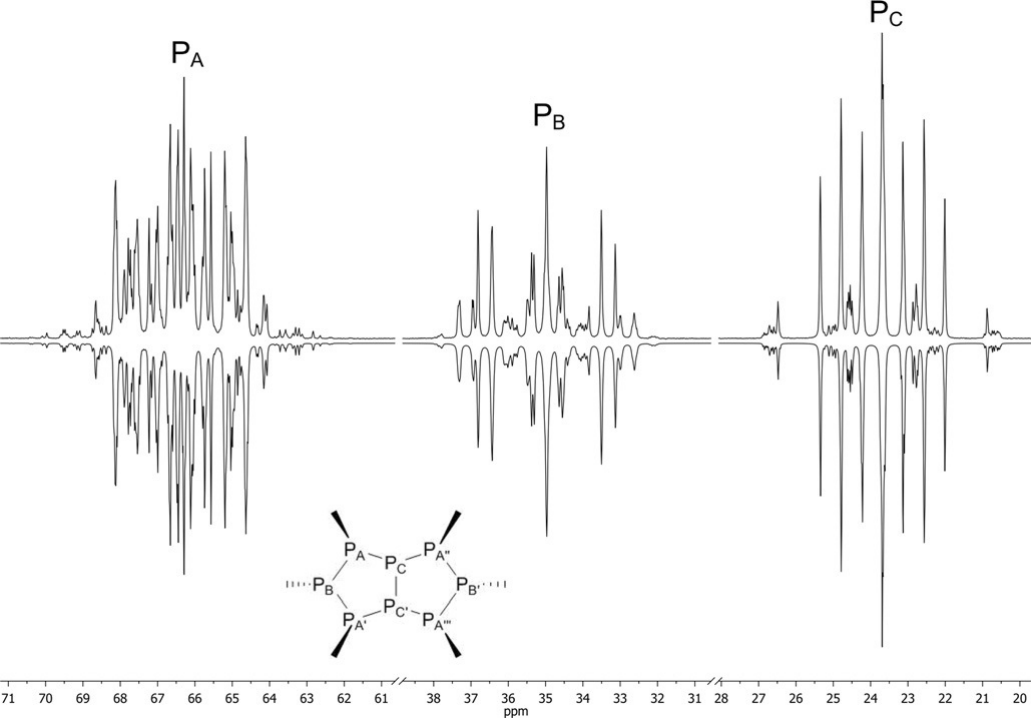
Experimental (top) and simulated (bottom) ^31^P{^1^H} NMR spectrum of octaphosphane **2** (AA′A′′A′′′BB′CC′ spin system with *C*_2*v*_ symmetry). Chemical shifts in ppm: *δ*(P_A_)=+66.3, *δ*(P_B_)=+35.0, *δ*(P_C_)=+23.7. Selected coupling constants in Hz: ^1^ *J*_AB_=−292.30(3), ^1^ *J*_AC_=−262.67(3), ^1^ *J*_CC′_=−311.99(12), ^2^ *J*_AA′′_=+143.18(8).

Clearly, there is a molecular process, which causes a higher effective symmetry in solution (*C*_2*v*_) compared to the solid state (*C*_2_). This was confirmed by VT NMR measurements, which showed that upon cooling, the signal of P_A_ in the ^31^P{^1^H} NMR spectrum (Figure S6, Supporting Information) appears to lose its mirror symmetry resulting in two partially overlapping multiplets. The situation is much clearer in the ^1^H{^31^P} NMR spectra (Figure S7, Supporting Information), in which a proper decoalescence of the singlet of the *t*Bu group attached to P_A_ (+1.38 ppm, *I*_rel_=2) was observed at −75 °C. The resulting pattern at lower temperatures is thus consistent with an AA′BB′CC′DD′ spin system (*C*_2_) matching the molecular structure in the solid state. The molecular motion responsible for the process is the libration around the bond between the bridgehead atoms (Scheme 3). We performed a relaxed surface scan of the respective dihedral angle (henceforth *λ*) by using DFT calculations (PBE0‐D3/def2‐TZVP; see Supporting Information). The profile (Figure S24, Supporting Information) indeed shows that the molecular structure in the solid state (*λ*=40.6°) presents a minimum on the energy‐surface potential, whereas the *C*_2*v*_‐symmetrical transition state (*λ*=0°) matches the average structure in solution. Furthermore, the respective activation energy of +32.6 kJ mol^−1^ agrees well with the experiment according to the temperature range, in which the coalescence is observed.

**Scheme 3:**
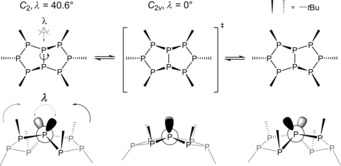
Libration of octaphosphane **2**. The transition state corresponds to the average structure in solution. The remaining structures (left and right) correspond to the enantiomeric structure found in the solid state.

In view of the discovery of octaphosphane **2**, which was stated to be unstable,^19^ the discovery of further octaphosphanes becomes more conceivable. We calculated the relative energies of all eight possible octanes C_8_H_14_, phosphanes P_8_H_6_, as well as organophosphanes P_8_ *t*Bu_6_ (Scheme 4, detailed in the Supporting Information) and draw some key conclusions from these calculations. Going from the octanes to the phosphanes (formal exchange of CH_2_ with PH) changes the order of relative energies, which is due to phosphorus preferring smaller bond angles than carbon. Replacing the hydrogen atom with *t*Bu groups changes the order once again for the organophosphanes. Structure **A** is usually considerably less stable than the other ones due to the higher strain of the four‐membered ring. However, attractive dispersion interactions between the *t*Bu groups stabilize this structure for the organophosphane **1**. For P_8_ *t*Bu_6_ thus type **A** (Δ*E*_rel_=28.2 kJ mol^−1^) and **B** (Δ*E*_rel_=0 kJ mol^−1^, octaphosphane **2**) are the most stable structures. Nevertheless, structures **D** and **F** are only slightly higher in energy (Δ*E*_rel_=41.1 and 60.4 kJ mol^−1^) and hence they are expected to be isolable compounds.

**Scheme 4:**
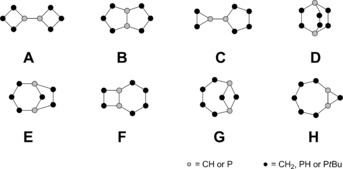
Possible constitutional isomers of C_8_H_14_, P_8_H_6_, or P_8_ *t*Bu_6_ precluding multiple bonds.

Like its constitutional isomer, octaphosphane **2** is able to coordinate gold(I). When **2** was reacted with one or two equivalents of [AuCl(tht)], a mononuclear complex [AuCl(**2**)] (**3**) or a dinuclear complex [(AuCl)_2_(**2**)] (**4**) was obtained, respectively (Scheme 5). The molecular structures of both complexes (Figures S19 and S20, Supporting Information) show that the peripheral phosphorus atoms of the ligand coordinate gold(I) chloride (κ*P* ^3^/κ*P* ^3′^ binding mode) resulting in a linear coordination environment with Au−P bond lengths as observed for similar complexes.^20^ The structural parameters of the ligand in **3** and **4** are not significantly different from the free octaphosphane **2** besides a slightly larger dihedral angle *λ*, which seems to be increasing with the number of AuCl fragments (44.8° for **3** and 50.69° for **4**). Mass spectrometry and elemental analysis confirm the composition of the proposed complexes.

**Scheme 5:**
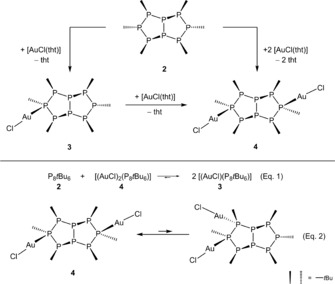
Formation and isomerization reaction of gold(I) complexes **3** and **4**.

However, ^31^P{^1^H} NMR spectroscopy indicates a more complex situation in solution. Thus, for **3**, five multiplets with a relative intensity (I_rel_) of 1:2:2:2:1 were observed, whereas the spectrum of **4** showed three multiplets with I_rel_ of 4:2:2. This is consistent with the respective solid‐state structure assuming the same libration motion as for the free octaphosphane equally causing a higher effective symmetry. Furthermore, in the ^31^P{^1^H} NMR spectrum of **3** (Figure S9, Supporting Information) additional signals corresponding to the free octaphosphane **2** and the bimetallic complex **4** were observed. This indicates an equilibrium in solution (Eq. (1), Scheme 5) analogous to the one observed for the gold(I) complexes of octaphosphane **1**.^9^ In fact, when octaphosphane **2** was reacted with the dinuclear complex **4**, the resulting NMR spectrum is identical to the one of complex **3**. Furthermore, this is also supported by the crystal structure of **3**, which co‐crystallized with 4 % of complex **4** (details in the Supporting Information). The situation is different for complex **4**. In the ^31^P{^1^H} NMR spectrum (Figure S12, Supporting Information), in addition to the signals which match the solid‐state structure, a second signal set was observed, for which we propose a constitutional isomer (Eq. (2), Scheme 5). Because the experimental PXR diffractogram of **4** matches well the theoretical pattern calculated from its single crystal structure data, it appears that the second isomer is only formed in solution.

The reaction of octaphosphane **2** with an excess of [AuCl(tht)] gave an insoluble substance, presumably, a coordination polymer. Elemental analysis indicates the formation of a tetranuclear complex [(AuCl)_4_(**2**)], and the IR spectrum showed the same characteristic vibrations of the *t*Bu group that were observed for **2**–**5**.

When octaphosphane **2** was reacted with an excess of *cis*‐[PdCl_2_(cod)] (cod=1,5‐cyclooctadiene), the chelate complex [PdCl_2_(**2**‐κ^2^ *P* ^2^,*P* ^2′^)] (**5**) was obtained (Scheme 6). The ligand features a different binding mode compared to the gold(I) complexes, now involving the phosphorus atoms (P_A_) adjacent to the bridgehead atoms in coordination (Figure 2). The coordination of the Pd atom (square‐planar coordination environment) furthermore induces some changes in the structure of the ligand. These include a very large dihedral angle *λ* (54.1°), as well as a significantly shorter bond between the bridgehead phosphorus atoms. The pattern of the ^31^P{^1^H} NMR spectrum of **5** (Figure S15, Supporting Information) agrees well with the expected AA′BB′CC′DD′ spin system (*C*_2_ symmetry). Remarkably, the change in the chemical shift induced by coordination is enormous. Thus, the coordinating atoms (P_A_) are deshielded by 90.1 ppm, whereas all other nuclei experience a high‐field shift by 45.1 (P_B_), 39.1 (P_C_), and even 89.0 ppm (P_D_) for the bridgehead atoms. All other analytical data, namely mass spectrometry, IR spectroscopy (characteristic *t*Bu vibrations), elemental analysis as well as ^1^H{^31^P} NMR (3 singlets) and ^13^C{^1^H,^31^P} NMR spectroscopy (6 singlets), are in excellent agreement with the observed structure.

**Scheme 6:**
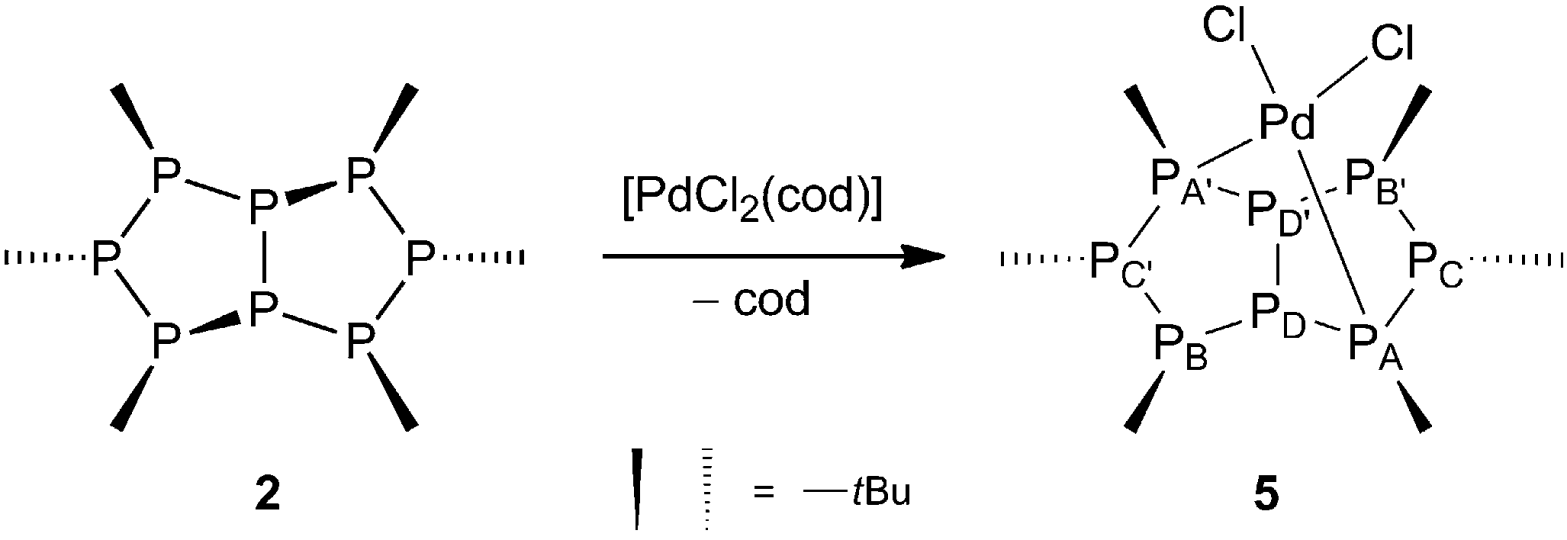
Reaction of octaphosphane **2** with *cis*‐[PdCl_2_(cod)].

**Figure 2:**
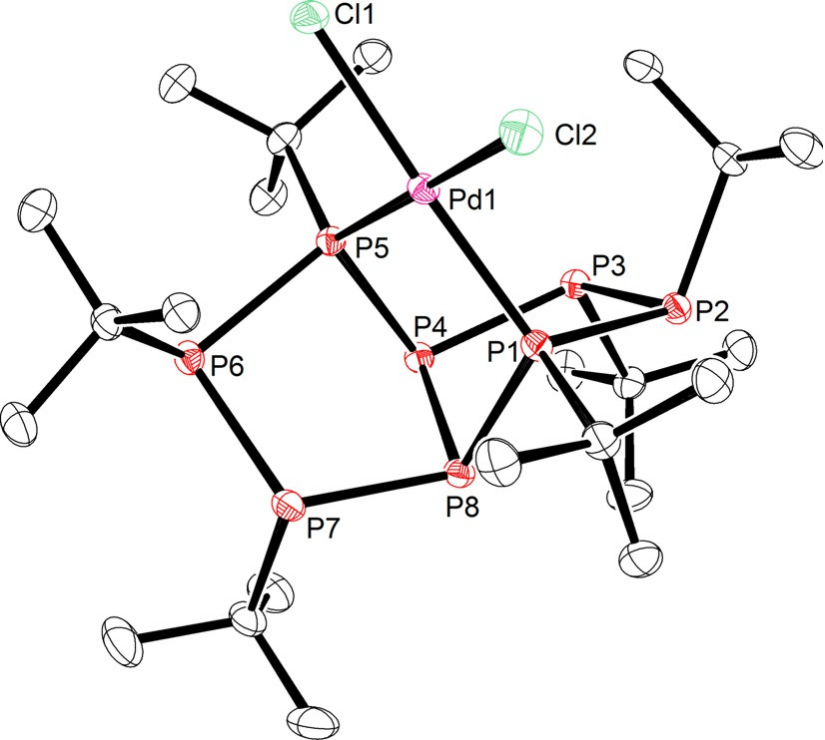
Molecular structure of complex **5** (H atoms and co‐crystallized solvent molecule omitted for clarity). Selected bond lengths [Å] and angles [°]: Pd1−P1 2.2657(4), Pd1−P5 2.2568(4), P4−P8 2.1564(5); P1‐Pd1‐P5 89.06(1), P1‐Pd1‐Cl2 90.89(1), P5‐Pd1‐Cl1 91.72(1), *λ* 54.1(1).

In conclusion, octaphosphane **2** exhibits a variety of different coordination modes, as shown in complexes **3**–**5**. The peripheral P atoms (P_C_ and equivalent) seem to be slightly better donors. However, chelation of a metal requires the coordination of the P atoms adjacent to the bridgehead atoms (P_A_ and equivalent). Discovery of additional coordination modes in the future are very likely. Further insights might also be gained preparing complexes with NMR‐active metals, such as ^103^Rh and ^195^Pt. These results once again show how complex the coordination chemistry of an apparently simple phosphane molecule can be.^9^ They furthermore underline the similarity between phosphorus‐rich compounds and their isolobal organic molecules.

## Conflict of interest

The authors declare no conflict of interest.

## Supporting information

As a service to our authors and readers, this journal provides supporting information supplied by the authors. Such materials are peer reviewed and may be re‐organized for online delivery, but are not copy‐edited or typeset. Technical support issues arising from supporting information (other than missing files) should be addressed to the authors.

Supplementary

## References

[1a] H. Köhler , A. Michaelis , Ber. Dtsch. Chem. Ges. 1877, 10, 807–814;

[1b] G. Boeck , T. Peppel , D. Selent , A. Schulz , Nachr. Chem. 2017, 65, 1030–1033.

[2a] M. Baudler , K. Glinka , Chem. Rev. 1993, 93, 1623–1667;

[2b] M. Baudler , K. Glinka , Chem. Rev. 1994, 94, 1273–1297.

[3a] M. Caporali , L. Gonsalvi , A. Rossin , M. Peruzzini , Chem. Rev. 2010, 110, 4178–4235; 20170154 10.1021/cr900349u

[3b] M. Scheer , G. Balázs , A. Seitz , Chem. Rev. 2010, 110, 4236–4256. 20438122 10.1021/cr100010e

[4] A. Kircali , R. Frank , S. Gómez-Ruiz , B. Kirchner , E. Hey-Hawkins , ChemPlusChem 2012, 77, 341–344.

[5a] A. Kırcalı Akdag , P. Lönnecke , E. Hey-Hawkins , Z. Anorg. Allg. Chem. 2014, 640, 271–274;

[5b] D. M. Yufanyi , T. Grell , M.-B. Sárosi , P. Lönnecke , E. Hey-Hawkins , Pure Appl. Chem. 2019, 91, 785–796.

[6] M. Yoshifuji , I. Shima , N. Inamoto , K. Hirotsu , T. Higuchi , J. Am. Chem. Soc. 1981, 103, 4587–4589.

[7] M. Baudler , S. Akpapoglou , D. Ouzounis , F. Wasgestian , B. Meinigke , H. Budzikiewicz , H. Münster , Angew. Chem. Int. Ed. Engl. 1988, 27, 280–281;

[8] K. B. Dillon , F. Mathey , J. F. Nixon , Phosphorus. The Carbon Copy; From Organophosphorus to Phospha-Organic Chemistry, Wiley, Chichester, 1998.

[9] T. Grell, E. Hey-Hawkins, *Eur. J. Inorg. Chem* **2019**, submitted.

[10a] A. Schisler , P. Lönnecke , E. Hey-Hawkins , Phosphorus Sulfur Silicon Relat. Elem. 2002, 177, 1447–1450;

[10b] S. Gómez-Ruiz , E. Hey-Hawkins , Coord. Chem. Rev. 2011, 255, 1360–1386;

[10c] S. Gómez-Ruiz , E. Hey-Hawkins , New J. Chem. 2010, 34, 1525–1532.

[11] M. Baudler , T. Pontzen , J. Hahn , H. Ternberger , W. Faber , Z. Naturforsch. B 1980, 35, 517–521.

[12] C. von Hänisch , D. Fenske , Z. Anorg. Allg. Chem. 1997, 623, 1040–1042.

[13] N. W. Tun , I. Y. Shchapin , A. I. Nekhaev , Pet. Chem. 2013, 53, 335–340.

[14a] M. Baudler , J. Hahn , V. Arndt , B. Koll , K. Kazmierczak , E. Därr , Z. Anorg. Allg. Chem. 1986, 538, 7–20;

[14b] M. Baudler , H. Jachow , Z. Anorg. Allg. Chem. 1990, 580, 27–35;

[14c] M. Baudler , H. Jachow , K.-F. Tebbe , Z. Anorg. Allg. Chem. 1991, 593, 9–16.

[15] M. Baudler , Y. Aktalay , Z. Anorg. Allg. Chem. 1983, 496, 29–39.

[16a] B. Riegel , A. Pfitzner , Y. G. Heckmann , H. Binder , E. Fluck , Z. Anorg. Allg. Chem. 1995, 621, 1365–1372;

[16b] M. Baudler , H. Tschäbunin , Z. Anorg. Allg. Chem. 1984, 511, 77–83;

[16c] R. Schmidt , S. A. Moya , D. Villagra , H. Binder , Y. G. Heckmann , Bol. Soc. Chil. Quim. 1996, 41, 371–375;

[16d] G. Fritz , J. Härer , K. Stoll , Z. Anorg. Allg. Chem. 1983, 504, 47–54.

[17] J. Hahn in Methods in Stereochemical Analysis, Vol. 8 (Ed.: J. G. Verkade), VCH, Weinheim, 1987.

[18] M. Bäudler , E. Därr , G. Binsch , D. S. Stephenson , Z. Naturforsch. B 1984, 39, 1671–1675.

[19] M. Baudler , J. Hellmann , P. Bachmann , K.-F. Tebbe , R. Fröhlich , M. Fehér , Angew. Chem. Int. Ed. Engl. 1981, 20, 406–408;

[20a] D. M. Stefanescu , H. F. Yuen , D. S. Glück , J. A. Golen , A. L. Rheingold , Angew. Chem. Int. Ed. 2003, 42, 1046–1048; 10.1002/anie.200390270 12616564

[20b] E. J. Fernández , M. C. Gimeno , P. G. Jones , A. Laguna , M. Laguna , J. M. Lopez-de-Luzuriaga , Angew. Chem. Int. Ed. Engl. 1994, 33, 87–88;

[20c] H. Schmidbaur , T. Pollok , G. Reber , G. Müller , Chem. Ber. 1988, 121, 1345–1348.

