# Supplementary material for: Unexpected Isomerization of Hexa‐tert‐butyl‐octaphosphane
Source: Chemistry. 2019 Dec 27;26(5):1008–12. doi: 10.1002/chem.201904531 (PMC7027762; doi:10.1002/chem.201904531)
Supplement: Supplementary file 1 — Supplementary [file CHEM-26-1008-s001.pdf]

CHEMISTRY
A European Journal

Supporting Information

Unexpected Isomerization of Hexa-*tert*-butyl-octaphosphane

Toni Grell and Evamarie Hey-Hawkins*[a]

chem_201904531_sm_miscellaneous_information.pdf

Abstract: Octaphosphane {*cyclo*-(P_4*t*Bu_3)}$_2$ (**1**) undergoes an unexpected isomerization reaction to the constitutional isomer 2,2',2'',2''',3,3'-hexa-*tert*-butyl-bicyclo[3.3.0]octaphosphane (**2**) in the presence of Lewis acidic metal salts. The mechanism of this reaction is discussed and elucidated with DFT calculations. The results underline the fascinating similarity between phosphorus-rich and isolobal carbon compounds. The new bicyclic octaphosphane **2** shows a dynamic behavior in solution and reacts with [AuCl(tht)] (tht = tetrahydrothiophene) to give a mono- ([AuCl(**2**-κP^3)], **3**) and a dinuclear complex ([$(AuCl)_2$(**2**-κP^3,κ$P^{3'}$)], **4**). With *cis*-[$PdCl_2$(cod)] (cod = 1,5-cyclooctadiene), the chelate complex ([$PdCl_2$(**2**-κ$^2P^2$,$P^{2'}$)], **5**) with a different coordination mode of the ligand was obtained.

1. Table of Contents

1. Table of Contents 2
2. Experimental Section 2
General Remarks 2
2,2',2'',2''',3,3'- Hexa-*tert*-butyl-bicyclo[3.3.0]octaphosphane (**2**) 3
Reaction Study of the Isomerization of Octaphosphanes **1** and **2** 4
Synthesis of [AuCl(**2**)] (**3**) 5
Synthesis of $[(AuCl)_2$(**2**)]·2THF (**4**·2THF) 5
Synthesis of Coordination Polymer $[(AuCl)_4$(**2**)$]_n$ 6
Reaction of Octaphosphane **2** with $[(AuCl)_2$(**2**)] (**3**) 6
Synthesis of $[PdCl_2$(**2**)] (**5**) 7
3. NMR Measurements and Spectra of the Compounds 8
NMR Spectra of 2,2',2'',2''',3,3'- Hexa-*tert*-butyl-bicyclo[3.3.0]octaphosphane (**2**) 8
NMR Spectra of [AuCl(**2**)] (**3**) 13
NMR Spectra of $[(AuCl)_2$(**2**)] (**4**) 15
NMR Spectra of $[PdCl_2$(**2**)] (**5**) 17
4. Mass spectrometry measurements 18
5. X-Ray Diffraction 19
Single Crystal Measurements 19
Powder X-ray Diffraction Measurements 23
6. Quantum Chemical Calculations 24
General Remarks 24
Benchmark Study 24
Librational Profile of Octaphosphane **2** 24
Mechanism of the Isomerization Reaction 25
Comparison of Isomers of E_8R_6 26
7. References 27

2. Experimental Section

General Remarks

All experiments were carried out under nitrogen using standard Schlenk techniques. Toluene, dichloromethane, diethyl ether, pentanes as well as hexanes (isomeric mixtures) and acetonitrile were dried and degassed with an MB SPS-800 Solvent Purification System (MBRAUN) and kept over molecular sieves (4 Å). THF was distilled from potassium and benzophenone and kept over molecular sieves (4 Å). The compounds {*cyclo*-($P_4{}^tBu_3$)}$_2$ (**1**)[1] and [AuCl(tht)][2] were prepared as described in the literature. All other chemicals were purchased and used without further purification.

Elemental Analysis. A VARIO EL (HERAEUS) microanalyzer was used to determine the elemental composition of the compounds.

Infra-red Spectroscopy. Samples were prepared as KBr pellets in a nitrogen-filled glove box and the infrared spectra were recorded in a range of 400 − 4000 cm^{-1}. A PERKIN-ELMER (System 2000) FT-IR spectrometer was used for compound **2** and a BRUKER (TENSOR 27) FT-IR spectrometer for compounds **3** and **4**. Abbreviation for labeling vibrations: w – weak; m – medium; s – strong; δ_s – symmetric deformation vibration; δ_{as} – asymmetric deformation vibration; ν_s – symmetric stretching vibration; ν_{as} – asymmetric stretching vibration; ρ – rocking vibration.

Luminescence. Luminescence was tested at 254 and 366 nm using a common UV lamp.

2,2',2'',2''',3,3'- Hexa-*tert*-butyl-bicyclo[3.3.0]octaphosphane (2)

15 mL of toluene were added to a mixture of octaphosphane **1** (82.3 mg, 139.4 mmol, 1.0 eq) and anhydrous $ZnCl_2$ (780 mg, 5723 mmol, 41 eq) and heated to reflux for 10 days. The mixture was filtered, and 5 mL of solvent were removed under reduced pressure. After one day at –15 °C colorless crystals of **1** formed. The solution was filtered and stored at –15 °C which gave colorless crystals of **2** after 3 days. These were washed with 2 mL of hexanes and dried in vacuum for 10 minutes.

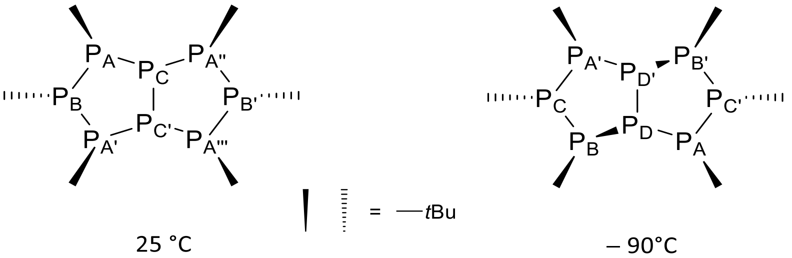

25 °C – 90°C

Yield: 62 mg (76%, colorless crystalline solid).

MP: 267 °C (Decomp.)

^{1}H{^{31}P} NMR (THF-*d*$_8$, 25 °C): δ = 1.44 (s, 18H, (**H**$_3$C)$_3$C-P$_B$), 1.38 (s, 36H, (**H**$_3$C)$_3$C-P$_A$) ppm.

^{13}C{^{1}H,^{31}P} NMR (THF-*d*$_8$): δ = 32.3 (s, **C**-P$_A$), 31.5 (s, (H$_3$**C**)$_3$C-CP$_A$), 30.2 (s, (H$_3$**C**)$_3$C-CP$_B$), 28.9 (s, **C**-P$_B$) ppm.

^{31}P{^{1}H} NMR (THF-*d*$_8$, 25 °C): AA'A''A'''BB'CC' spin system (C_{2V} symmetry):

**Chemical shifts [ppm]**		**Linewidth [Hz]**	**R-Factor**
δ_A = +66.3350(1)		LW(A) = 4.26(3)	0.9353%
δ_B = +35.0192(2)		LW(B) = 6.89(8)	
δ_C = +23.7065(1)		LW(C) = 3.64(4)	
**Coupling Constants [Hz]**			
$^2J_{AA'} = {}^2J_{A''A'''}$ =	–12.99(5)		
$^2J_{AA''} = {}^2J_{A'A'''}$ =	+143.18(8)		
$^3J_{AA'''} = {}^2J_{A'A''}$ =	+6.21(3)		
$^1J_{AB} = {}^1J_{A'B} = {}^1J_{A''B'} = {}^1J_{A'''B'}$ =	–292.30(3)		
$^3J_{AB'} = {}^3J_{A'B'} = {}^3J_{A''B} = {}^3J_{A'''B}$ =	–6.12(3)		
$^1J_{AC} = {}^1J_{A'C'} = {}^1J_{A''C} = {}^1J_{A'''C'}$ =	–262.67(3)		
$^2J_{AC'} = {}^2J_{A'C} = {}^2J_{A''C'} = {}^2J_{A'''C}$ =	–7.53(3)		
$^1J_{BB'}$ =	–1.45(5)		
$^2J_{BC} = {}^2J_{BC'} = {}^2J_{B'C} = {}^2J_{B'C'}$ =	+2.67(3)		
$^1J_{CC'}$ =	–311.99(12)		

^{1}H{^{31}P} NMR (THF-*d*$_8$, –90 °C): δ = 1.41 (s, 18H, (**H**$_3$C)$_3$C-P$_C$), 1.39 (s, 18H, (**H**$_3$C)$_3$C-P$_A$), 1.32 (s, 18H, (**H**$_3$C)$_3$C-P$_B$) ppm.

^{31}P{^{1}H} NMR (THF-*d*$_8$, –90 °C): δ = 64.0 (m, 2P, P$_A$), 62.6 (m, 2P, P$_B$), 32.1 (m, 2P, P$_C$), 23.1 (m, 2P, P$_D$) ppm.

IR (KBr): $\tilde{\nu}$ = 3187 (w), 2931 (s, ν_{as}(C–H)), 2886 (s, ν_s(C–H)), 2705 (w), 1455 (s, δ_{as}(CH$_3$)), 1385 (m, δ_s(CH$_3$)), 1357 (s, δ_s(CH$_3$)), 1167 (s, ρ(CH$_3$) + ν_s(C–C)), 1010 (m, ρ(CH$_3$)), 934 (m, ρ(CH$_3$) + ν_s(C–C)), 805 (m, ν_s(C–C)), 727 (w), 694 (w), 571 (w), 490 (w), 464 (w) cm^{-1}.

MS (ESI(+), MeCN/THF 1:1): *m/z* = 239.0 [P$_4{}^t$Bu$_2$H]$^+$ (1%), 255.0 [P$_4{}^t$Bu$_2$OH]$^+$ (1%), 271.0 [P$_4{}^t$Bu$_2$O$_2$H]$^+$ (1%), 287.0 [P$_4{}^t$Bu$_2$O$_3$H]$^+$ (1%), 303.0 [P$_4{}^t$Bu$_2$O$_4$H]$^+$ (1%), 327.1 [P$_5{}^t$Bu$_3$H]$^+$ (1%), 343.1 [P$_5{}^t$Bu$_3$OH]$^+$ (3%), 359.1 [P$_5{}^t$Bu$_3$O$_2$H]$^+$ (2%), 375.1 [P$_5{}^t$Bu$_3$O$_3$H]$^+$ (2%), 415.1 [P$_6{}^t$Bu$_4$H]$^+$ (2%), 431.1 [P$_6{}^t$Bu$_4$OH]$^+$ (2%), 447.1 [P$_6{}^t$Bu$_4$O$_2$H]$^+$ (2%), 463.1 [P$_6{}^t$Bu$_4$O$_3$H]$^+$ (7%), 479.1 [P$_6{}^t$Bu$_4$O$_4$H]$^+$ (2%), 495.1 [P$_6{}^t$Bu$_4$O$_5$H]$^+$ (2%), 503.2 [P$_7{}^t$Bu$_5$H]$^+$ (2%), 519.2 [P$_7{}^t$Bu$_5$O$_1$H]$^+$ (10%), 535.2 [P$_7{}^t$Bu$_5$O$_2$H]$^+$ (5%), 551.2 [P$_7{}^t$Bu$_5$O$_3$H]$^+$ (3%), 567.2 [P$_7{}^t$Bu$_5$O$_4$H]$^+$ (5%), 583.1 [P$_7{}^t$Bu$_5$O$_5$H]$^+$ (20%), 599.1 [P$_7{}^t$Bu$_5$O$_6$H]$^+$ (11%), 607.2 [P$_8{}^t$Bu$_6$OH]$^+$ (18%), 615.1 [P$_7{}^t$Bu$_5$O$_7$H]$^+$ (1%), 623.2 [P$_8{}^t$Bu$_6$O$_2$H]$^+$ (100%), 629.2 [P$_8{}^t$Bu$_6$ONa]$^+$ (3%), 639.2 [P$_8{}^t$Bu$_6$O$_3$H]$^+$ (77%), 645.2 [P$_8{}^t$Bu$_6$O$_2$Na]$^+$ (17%), 655.2 [P$_8{}^t$Bu$_6$O$_4$H]$^+$ (26%), 661.2

$[P_8{}^tBu_6O_3Na]^+$ (29%), 671.2 $[P_8{}^tBu_6O_5H]^+$ (5%), 677.2 $[P_8{}^tBu_6O_4Na]^+$ (17%), 693.2 $[P_8{}^tBu_6O_5Na]^+$ (4%), 709.2 $[P_8{}^tBu_6O_6Na]^+$ (1%), 1147.4 $[(P_8{}^tBu_6)(P_7{}^tBu_5)O_2Na]^+$ (1%), 1163.4 $[(P_8{}^tBu_6)(P_7{}^tBu_5)O_3Na]^+$ (2%), 1179.4 $[(P_8{}^tBu_6)(P_7{}^tBu_5)O_4Na]^+$ (1%), 1195.4 $[(P_8{}^tBu_6)(P_7{}^tBu_5)O_5Na]^+$ (1%), 1211.4 $[(P_8{}^tBu_6)(P_7{}^tBu_5)O_6Na]^+$ (1%), 1251.4 $[(P_8{}^tBu_6)_2O_3Na]^+$ (1%), 1267.4 $[(P_8{}^tBu_6)_2O_4Na]^+$ (4%), 1283.4 $[(P_8{}^tBu_6)_2O_5Na]^+$ (11%), 1299.4 $[(P_8{}^tBu_6)_2O_6Na]^+$ (14%), 1315.4 $[(P_8{}^tBu_6)_2O_7Na]^+$ (10%), 1331.4 $[(P_8{}^tBu_6)_2O_8Na]^+$ (3%), 1347.4 $[(P_8{}^tBu_6)_2O_9Na]^+$ (1%).

**Elemental Analysis** for $C_{24}H_{54}P_8$	Found (%)	C: 49.04	H: 9.56
	Calculated (%)	C: 48.82	H: 9.22

Reaction Study of the Isomerization of Octaphosphanes 1 and 2

The reactions were carried out in sealed glass NMR tubes. The respective octaphosphane and $ZnCl_2$ were added and evacuated, then 0.6 mL C_6D_6 were added. The suspension was kept at 70 °C in a thermostat without stirring. For the reaction with 0.1 equivalents of $ZnCl_2$, the right amount of $ZnCl_2$ was added by preparing a stock solution of $ZnCl_2$ in water, adding this to the NMR tube and evaporating the solvent. The composition of the reaction mixture was determined based on the relative intensity of all ^{t}Bu protons from $^1H\{^{31}P\}$ NMR spectroscopy. Heating was stopped for approximately 30 minutes for each measurement. The amounts of the starting materials in the different reactions were:

Phosphane **1** (16.6 mg, 1.0 eq), $ZnCl_2$ (0.3 mg, 0.1 eq)

Phosphane **1** (16.3 mg, 1.0 eq), $ZnCl_2$ (3.0 mg, 0.8 eq)

Phosphane **1** (18.4 mg, 1.0 eq), $ZnCl_2$ (23.0 mg, 5.4 eq)

Phosphane **2** (21.8 mg, 1.0 eq), $ZnCl_2$ (12.3 mg, 2.4 eq).

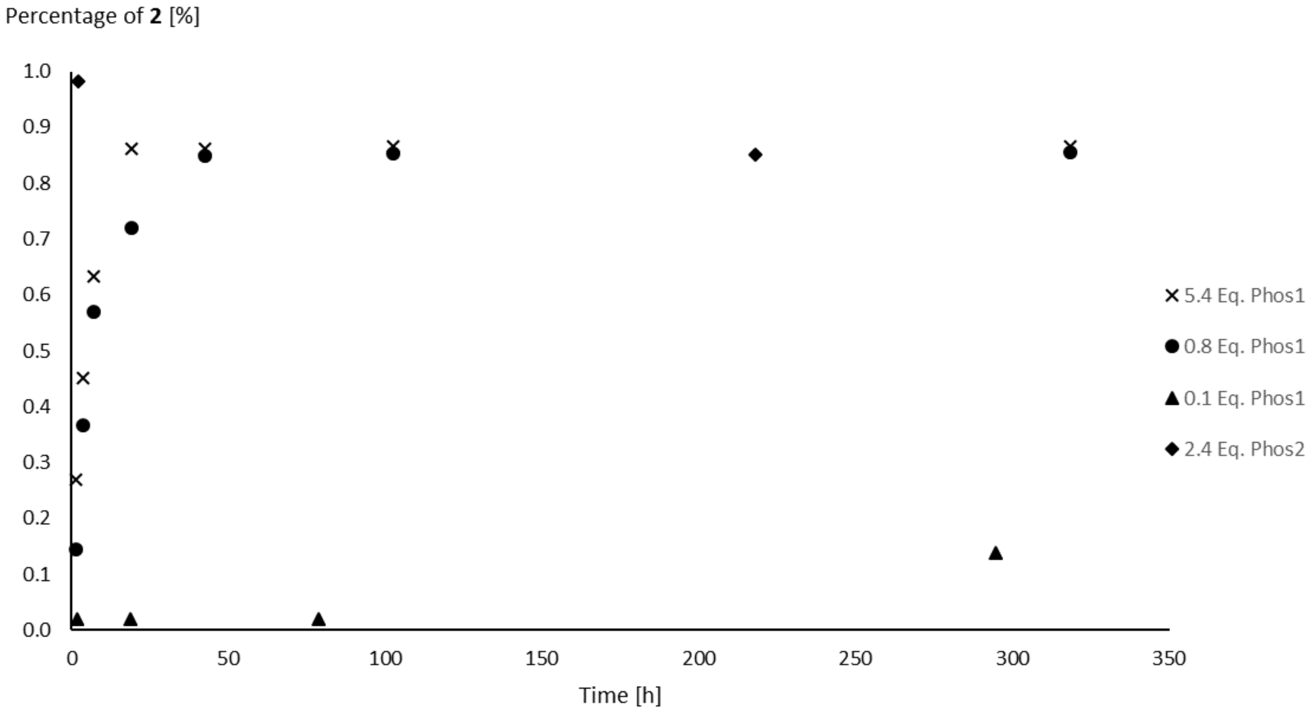

Figure S1. Results of the reaction study.

Synthesis of [AuCl(2)] (3)

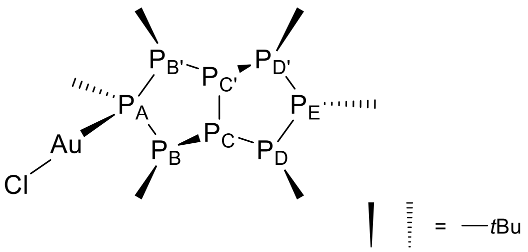

A solution of [AuCl(tht)] (26 mg, 81.1 mmol, 1.0 eq) in 8 mL THF was added dropwise to a solution of octaphosphane **2** (47 mg,79.6 mmol, 1.0 eq) in 7 mL THF resulting in a pale yellow solution. Evaporation of the solvent under reduced pressure gave a pale yellow powder. The compound showed no luminescent behavior.

Yield: 63 mg (96%).

MP: 217 °C

^{1}H{^{31}P} NMR (THF-*d*$_8$): δ = 1.55 – 1.20 (various s, C**H**$_3$) ppm.

^{13}C{^{1}H,^{31}P} NMR (THF-*d*$_8$,): δ = 35.5 – 1.20 (various s, CH$_3$ and **C**P) ppm.

^{31}P{^{1}H} NMR (THF-*d*$_8$): δ = 89.1 (m, 1P, P_A), 73.9 (m, 2P, P_B), 61.8 (m, 2P, P_C), 44.5 (m, 2P, P_D), 7.4 (m, 1P, P_E) ppm.

IR (KBr): $\tilde{\nu}$ = 3459(m), 2944 (s, ν_{as}(C–H)), 2886 (s, ν_s(C–H)), 2854 (s, ν_s(C–H)), 1634 (w), 1459 (s, δ_{as}(CH$_3$)), 1388 (m, δ_s(CH$_3$)), 1361 (s, δ_s(CH$_3$)), 1261 (w), 1166 (s, ρ(CH$_3$) + ν_s(C–C)), 1105 (s), 1012 (w, ρ(CH$_3$), 934 (w, ρ(CH$_3$) + ν_s(C–C)), 804 (m, ν_s(C–C)), 571 (w), 516 (w) cm^{-1}.

MS (ESI(+), MeCN): *m/z* = 787.2 [LAu]$^+$ (100%). L = $C_{24}H_{54}P_8$.

**Elemental Analysis** for $C_{24}H_{54}AuClP_8$	Found (%)	C: 35.00	H: 6.89
	Calculated (%)	C: 35.03	H: 6.61

Synthesis of [(AuCl)$_2$(2)]·2THF (4·2THF)

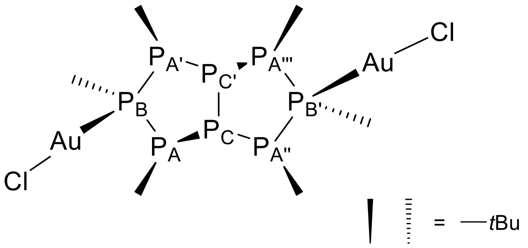

A solution of [AuCl(tht)] (58 mg, 180.9 mmol, 2.0 eq) in 8 mL THF was added dropwise to a solution of octaphosphane **2** (53 mg, 89.9 mmol, 1.0 eq) in 7 mL THF resulting in a deep yellow solution. Subsequently, the solvent was evaporated under reduced pressure, the residue dissolved in 3 mL THF and carefully layered with 12 mL of hexanes. After three days at 3 °C, large colorless crystals had formed which were isolated by decantation, washed with 2 mL of hexanes and dried in vacuum. The compound showed no luminescent behavior. The crystals can be dissolved in THF and the solvent removed under reduced pressure yielding the solvent free complex as a colorless powder.

Yield: 87 mg (80%).

MP: 225 °C

^{1}H{^{31}P} NMR (THF-*d*$_8$): δ = 1.42 – 1.20 (various s, C**H**$_3$) ppm.

^{13}C{^{1}H,^{31}P} NMR (THF-*d*$_8$): δ = 32.1 (s, (H$_3$**C**)$_3$C-CP$_A$), 31.4 (s, (H$_3$**C**)$_3$C-CP$_B$), 30.7 (s, (H$_3$**C**)$_3$C-CP$_C$) ppm. Quaternary carbon atoms could not be detected.

^{31}P{^{1}H} NMR (THF-*d*$_8$): δ = 52.4 (m, 4P, P_A), 50.3 (m, 2P, P_B) –9.9 (m, 2P, P_C) ppm.

IR (KBr): $\tilde{\nu}$ = 3446 (s), 2951 (s, ν_{as}(C–H)), 2888 (s, ν_s(C–H)), 2854 (s, ν_s(C–H)), 1636 (m), 1459 (s, $\delta_{as}(CH_3)$), 1390 (m, $\delta_s(CH_3)$), 1361 (s, $\delta_s(CH_3)$), 1261 (w), 1161 (s, $\rho(CH_3)$ + ν_s(C–C)), 1012 (m, $\rho(CH_3)$, 988 (m), 937 (w, $\rho(CH_3)$ + ν_s(C–C)), 802 (m, ν_s(C–C)), 657 (w), 582 (w), 521 (w), 463 (w) cm^{-1}.

MS (ESI(+), MeCN): *m/z* = 787.2 $[LAu]^+$ (18%), 1019.1 $[LAu_2Cl]^+$ (100%), 1077.1 $[LAu_2Cl_2Na]^+$ (22%), 1609.3 $[L_2Au_2Cl]^+$ (8%), 1841.3 $[L_2Au_2ClO]^+$ (16%). L = $C_{24}H_{54}P_8$.

**Elemental Analysis** for $C_{32}H_{70}Au_2Cl_2O_2P_8$	Found (%)	C: 32.20	H: 6.21
	Calculated (%)	C: 32.04	H: 5.88

Synthesis of Coordination Polymer $[(AuCl)_4(2)]_n$

15 mL of THF were added to a mixture of [AuCl(tht)] (90 mg, 280.1 mmol, 3.9 eq) in 8 mL THF and octaphosphane **2** (43 mg, 72.3 mmol, 1.0 eq) resulting in a white suspension. The reaction mixture was stirred overnight and filtered. A white precipitate formed which was filtered off and dried in vacuum. The precipitate showed no solubility in any common solvent.

MP: 256 °C

IR (KBr): $\tilde{\nu}$ = 3446 (s), 2953 (s, ν_{as}(C–H)), 2891 (s, ν_s(C–H)), 2856 (s, ν_s(C–H)), 1624 (m), 1457 (s, $\delta_{as}(CH_3)$), 1391 (m, $\delta_s(CH_3)$), 1362 (s, $\delta_s(CH_3)$), 1265 (m), 1165 (s, $\rho(CH_3)$ + ν_s(C–C)), 1098 (m), 1011 (m, $\rho(CH_3)$, 988 (m), 936 (w, $\rho(CH_3)$ + ν_s(C–C)), 802 (m, ν_s(C–C)), 716 (w), 576 (w), 529 (w), 501 (m) cm^{-1}.

**Elemental Analysis** for $C_{24}H_{54}Au_4Cl_4P_8$	Found (%)	C: 19.17	H: 3.35
	Calculated (%)	C: 18.96	H: 3.58

Reaction of Octaphosphane 2 with $[(AuCl)_2(2)]$ (3)

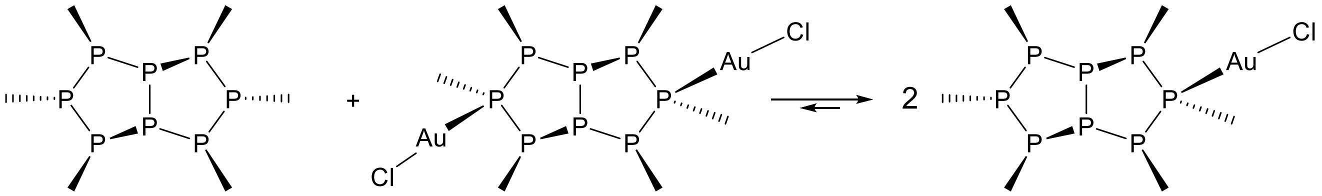

2 **4** = —*t*Bu **3**

10 mL of THF were added to a mixture of octaphosphane **2** (12 mg, 20 mmol, 1.0 eq) and complex of **4** (22 mg, 20 mmol, 1.0 eq). After 5 minutes, the suspension became clear and turned pale yellow. The solvent was removed under reduced pressure, the resulting residue dried in vacuum and investigated with NMR spectroscopy. The substance was analytically identical to **3**.

Synthesis of [$PdCl_2$(2)] (5)

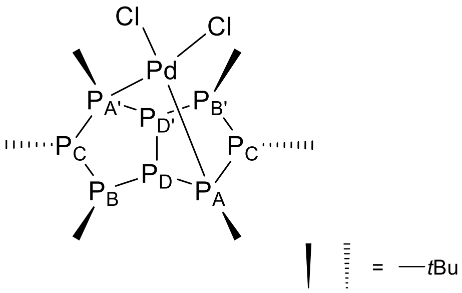

1,4-Dioxane (5 mL) was added to a mixture of [$PdCl_2$(cod)] (68.9 mg, 241.3 mmol, 3.3 eq) and octaphosphane **2** (43.8 mg, 74.1 mmol, 1.0 eq). Heating to reflux for 2 h resulted in a deep red solution. After cooling to rt, the solvent was removed under reduced pressure, the residue washed twice with 10 mL hexanes each and extracted 3 times with 5 mL toluene each. Evaporation of the solvent of the combined phases lead to a brown powder, which was dried in vacuum for 15 minutes.

Yield: 15.3 mg (27%).

MP: 247 °C

^{1}H{^{31}P} NMR (C_6D_6): δ = 1.93 (s, 18H, (**H**$_3$C)$_3$C-P_A), 1.64 (s, 18H, (**H**$_3$C)$_3$C-P_B), 1.10 (s, 18H, (**H**$_3$C)$_3$C-P_C) ppm.

^{13}C{^{1}H,^{31}P} NMR (C_6D_6): δ = 45.9 (s, (H_3C)$_3$**C**-CP_A), 35.2 (s, (H_3C)$_3$**C**-CP_B), 33.7 (s, (H_3C)$_3$**C**-CP_C) ppm, 32.32 (s, (H_3**C**)$_3$C-CP_A), 32.26 (s, (H_3**C**)$_3$C-CP_B), 31.2 (s, (H_3**C**)$_3$C-CP_C) ppm.

^{31}P{^{1}H} NMR (C_6D_6): δ = 156.4 (m, 2P, P_A), 21.2 (m, 2P, P_B), –4.3 (m, 2P, P_C),–66.3 (m, 2P, P_D) ppm.

IR (KBr): $\tilde{\nu}$ = 3450(m), 2958 (s, ν_{as}(C–H)), 2886 (s, ν_s(C–H)), 2855 (s, ν_s(C–H)), 1634 (w), 1459 (s, δ_{as}(CH_3)), 1389 (m, δ_s(CH_3)), 1362 (s, δ_s(CH_3)), 1261 (w), 1164 (s, ρ(CH_3) + ν_s(C–C)), 1099 (s), 1022 (w, ρ(CH_3), 938 (w, ρ(CH_3) + ν_s(C–C)), 803 (m, ν_s(C–C)), 667 (w), 585 (w), 525 (w), 497 (w), 423 (w) cm^{-1}.

MS (ESI(+), MeCN/C_6D_6 9:1): *m/z* = 731.1 [LPdCl]$^+$ (100%). L = $C_{24}H_{54}P_8$.

**Elemental Analysis** for $C_{24}H_{54}PdCl_2P_8$	Found (%)	C: 37.97	H: 6.58
	Calculated (%)	C: 37.54	H: 7.10

3. NMR Measurements and Spectra of the Compounds

NMR spectra were recorded at 25 °C with a BRUKER AVANCE DRX 400 spectrometer (^{1}H NMR: 400.13 MHz, ^{13}C NMR: 100.16 MHz, ^{31}P NMR: 161.97 MHz). TMS was used as internal standard for ^{1}H NMR spectroscopy. ^{13}C and ^{31}P NMR experiments were referenced to the Ξ scale.[3] Every signal labeling in this publication also extends to the chemically equivalent yet magnetically nonequivalent nuclei *i.e.* P_A for P_A, $P_{A'}$ as well as $P_{A''}$ and so forth. The ^{31}P NMR signals are labeled alphabetically starting from the nucleus which is most deshielded. All ^{31}P NMR spectra were measured with a 90° pulse and reduced acquisition (0.6 s) as well as d1 time (1.0 s). At least 1000 scans were necessary to obtain acceptable spectra. The samples were usually saturated solutions of the respective compound. Suitable spectra for simulation required about 100,000 scans. Simulation of the NMR spectra was performed using the DAISY module implemented in the program TopSpin version 3.6.1 (BRUKER, BioSpin GmbH, Rheinstetten). For this, the $^{1}J_{PP}$ coupling constants were set negative.[4]

NMR Spectra of 2,2',2'',2''',3,3'- Hexa-*tert*-butyl-bicyclo[3.3.0]octaphosphane (2)

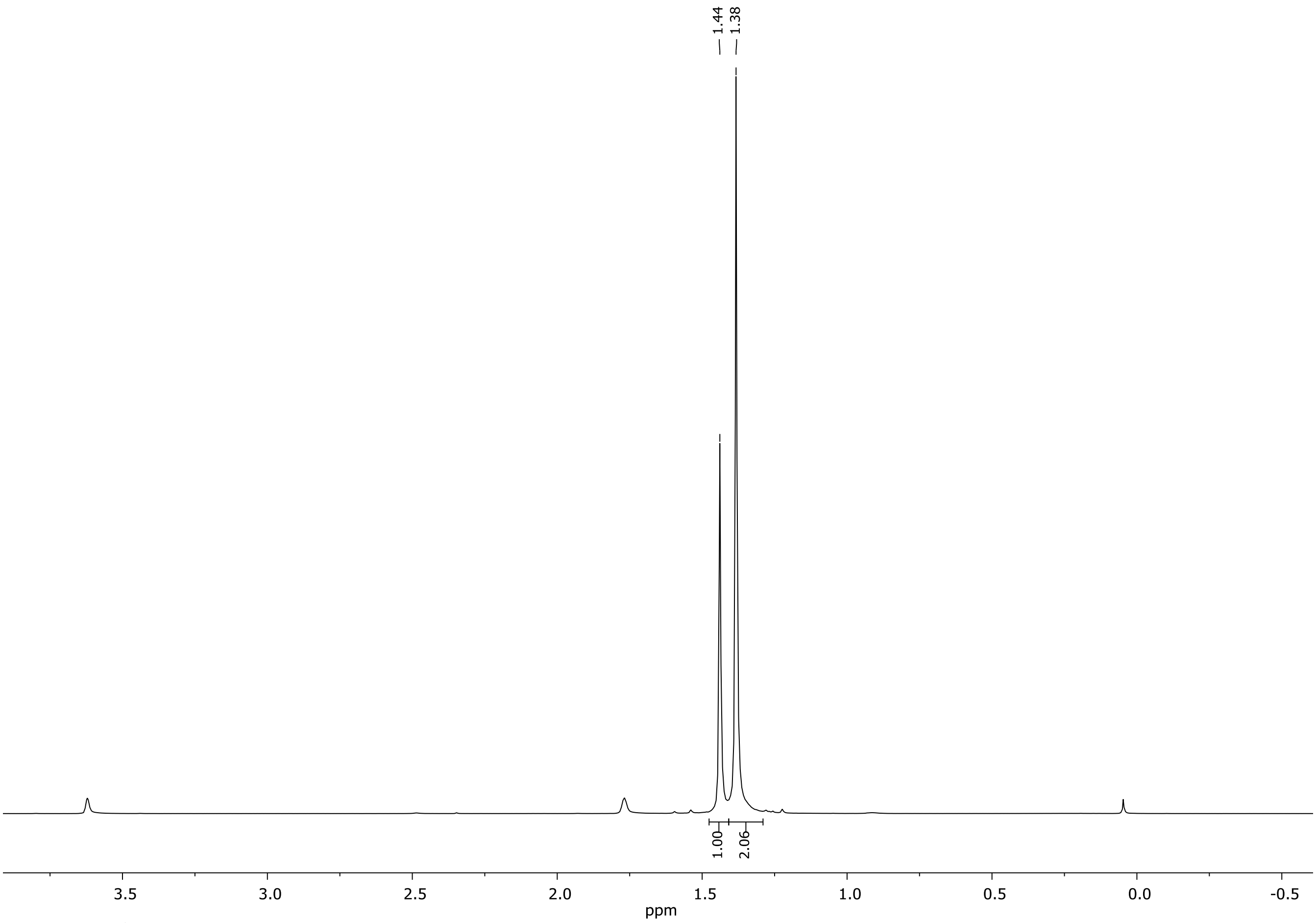

Figure S2. $^{1}H\{^{31}P\}$ NMR spectrum of octaphosphane **2** in THF-d_8.

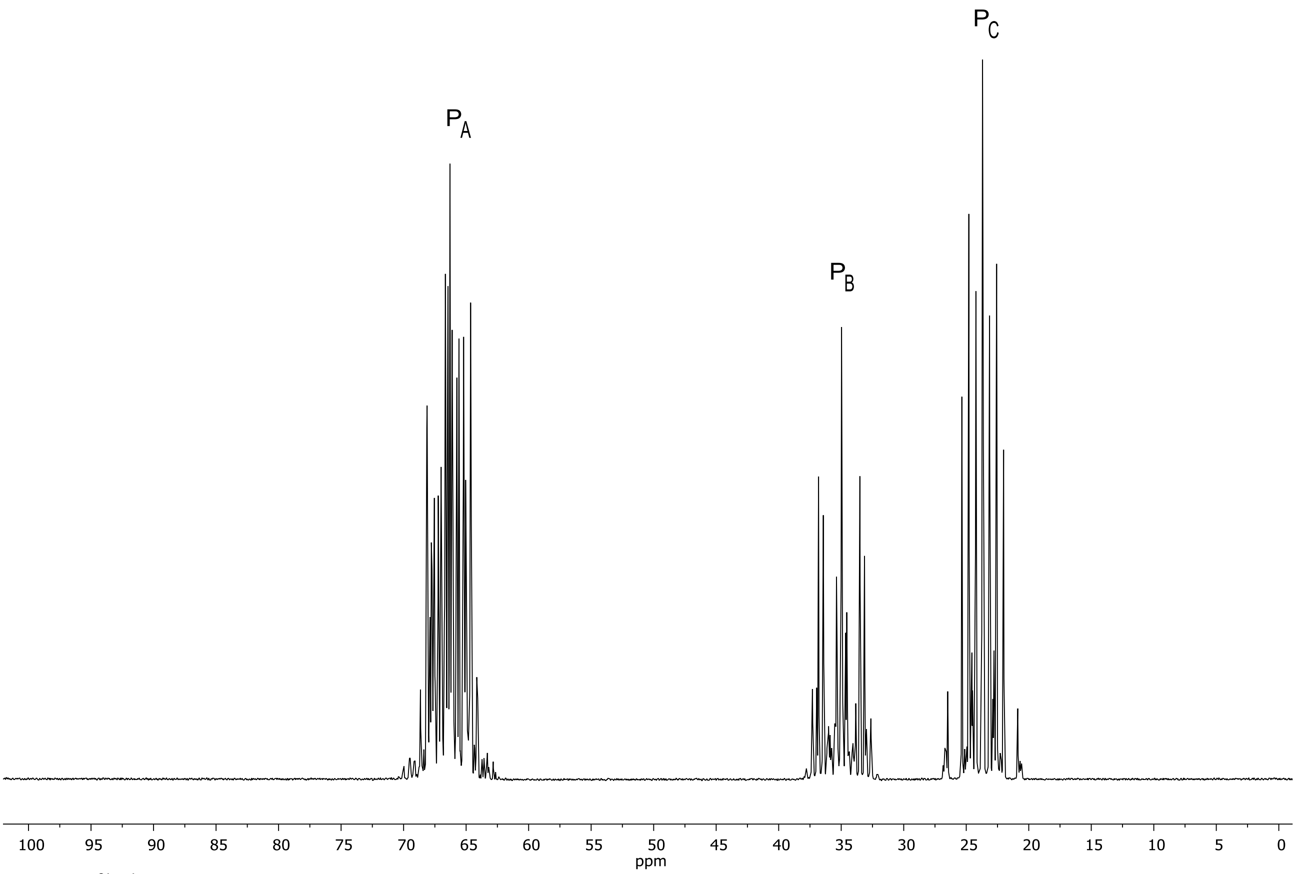

Figure S3. $^{31}P\{^{1}H\}$ NMR spectrum of octaphosphane **2** in THF-*d*$_8$.

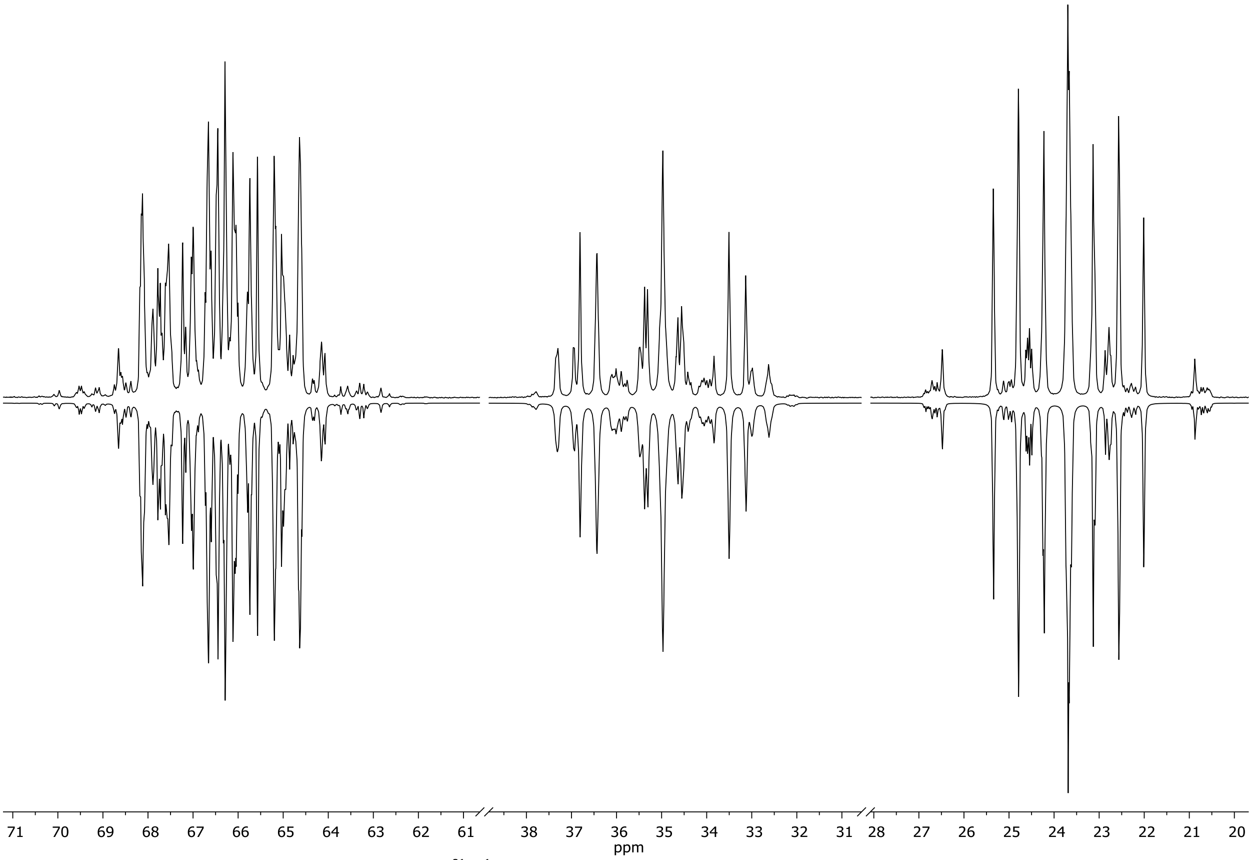

Figure S4. Experimental (top) and simulated (bottom) $^{31}P\{^{1}H\}$ NMR spectrum of octaphosphane **2** in THF-*d*$_8$.

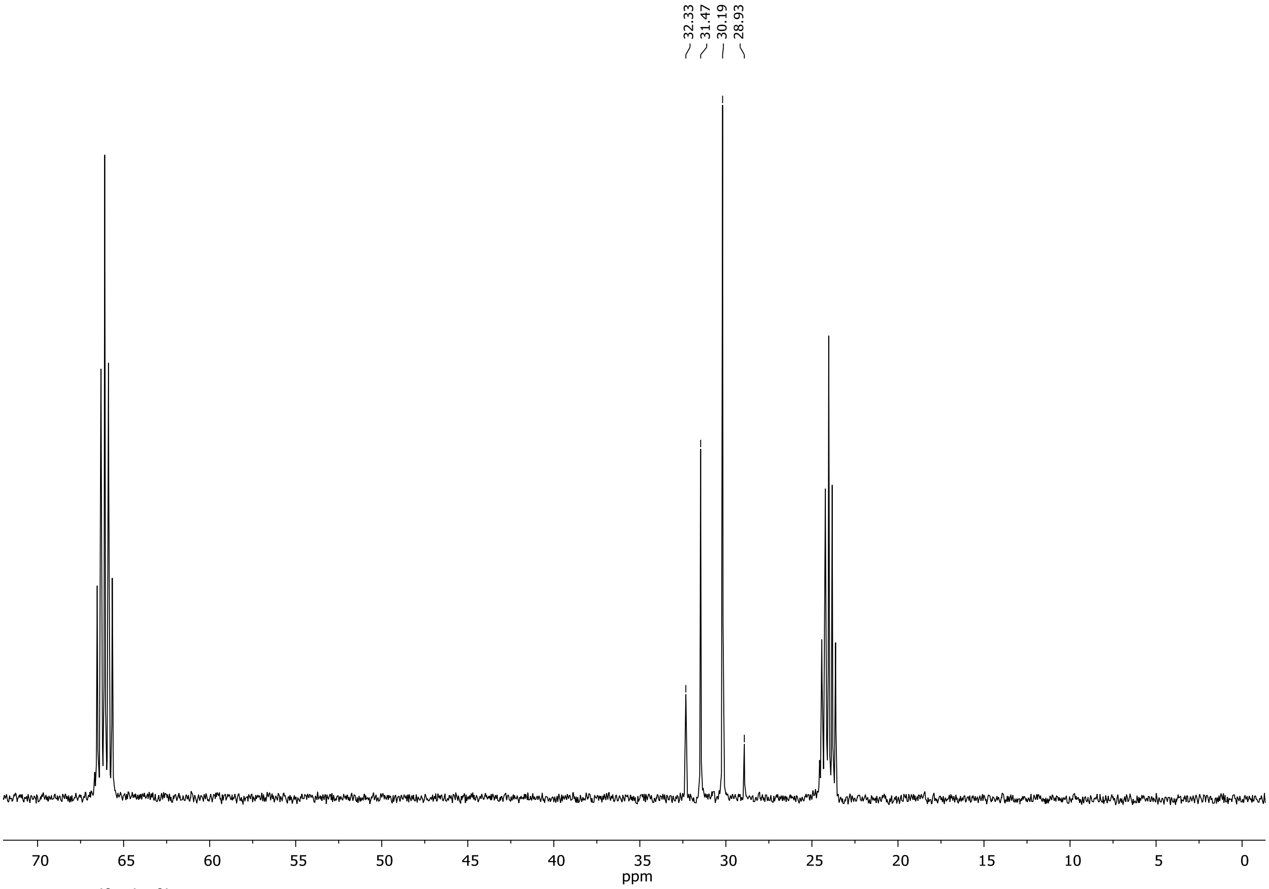

Figure S5. $^{13}C\{^{1}H,^{31}P\}$ NMR spectrum of octaphosphane **2** in C_6D_6.

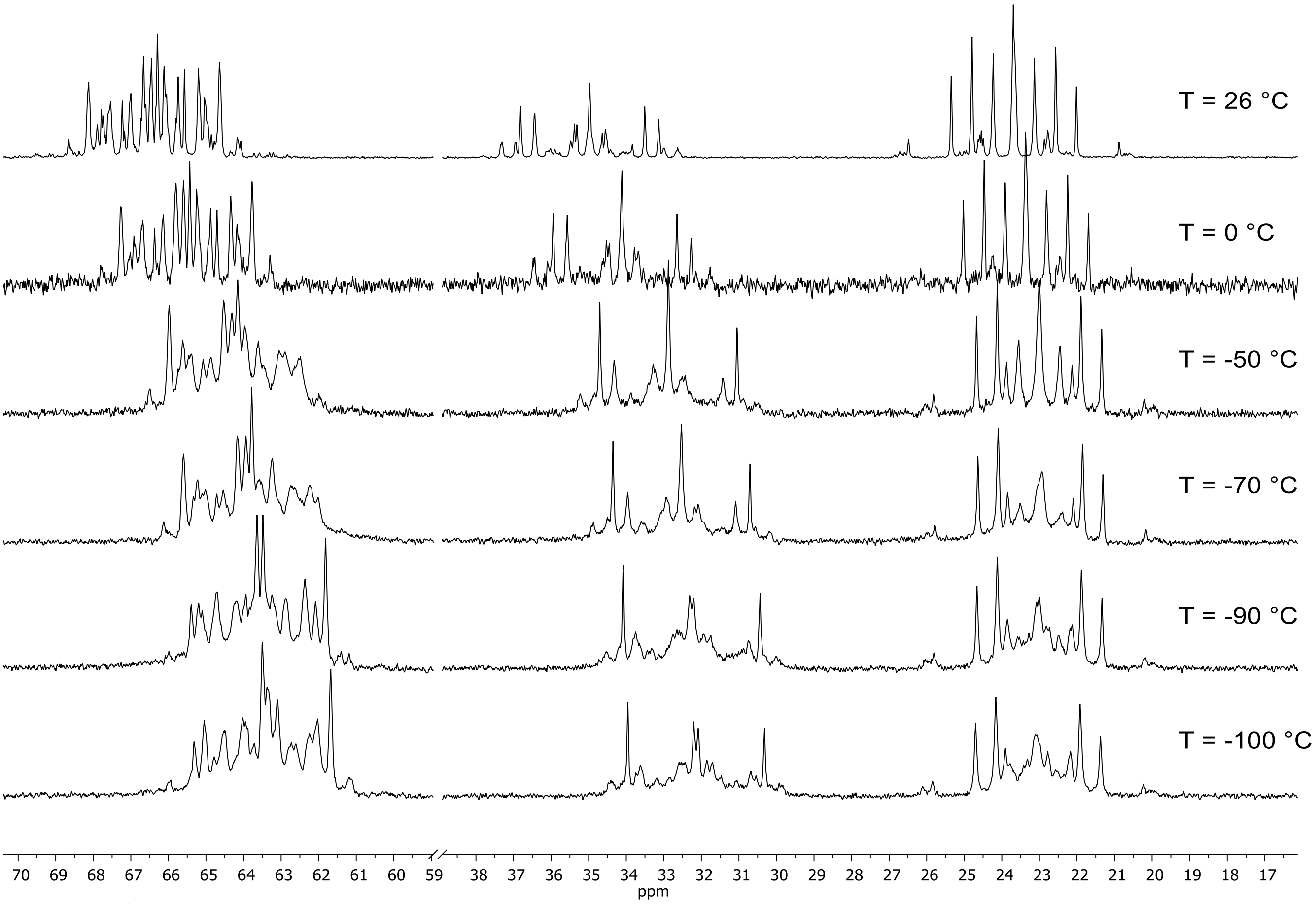

Figure S6. VT $^{31}P\{^{1}H\}$ NMR spectrum of octaphosphane **2** in THF-d_8.

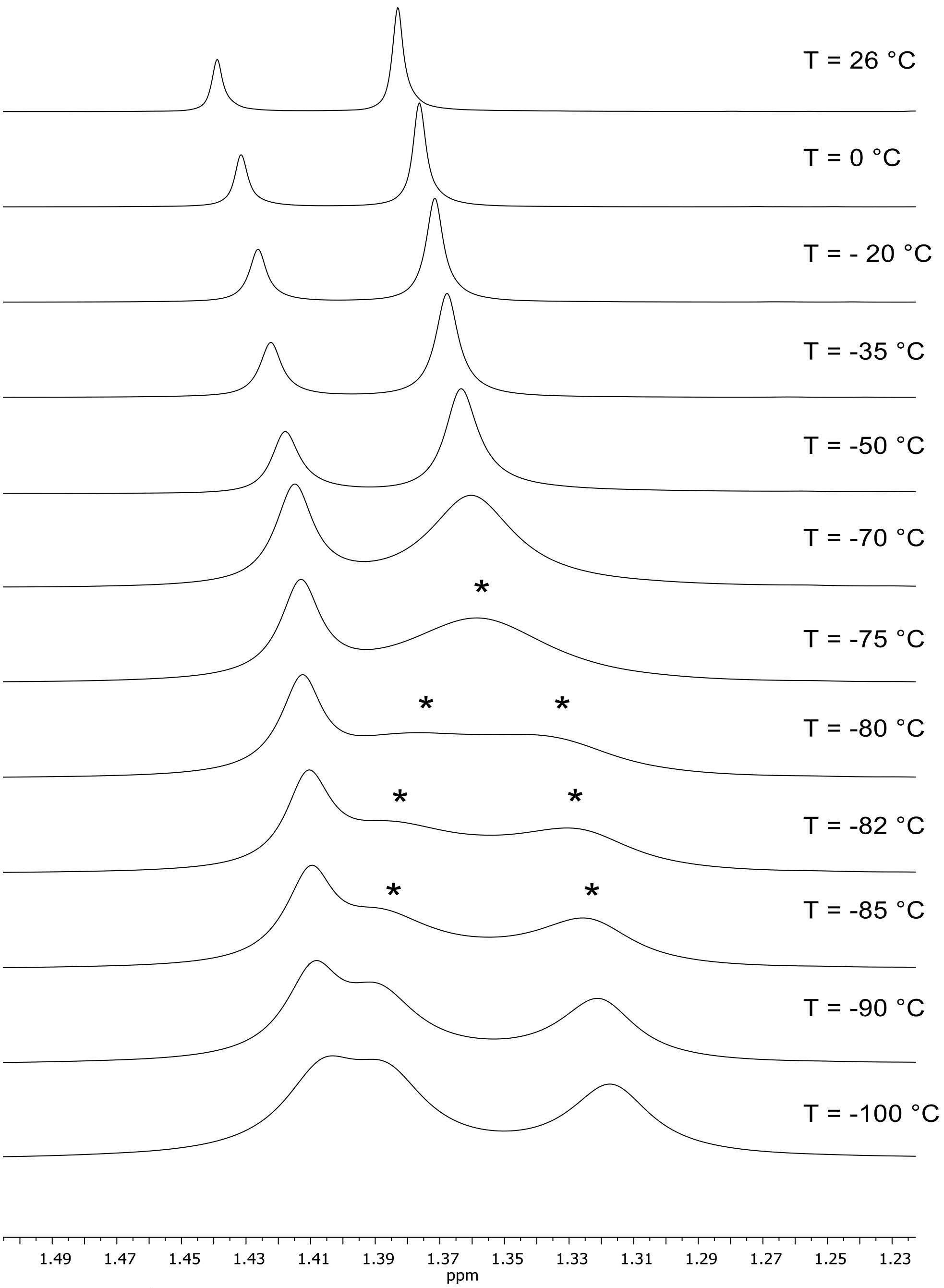

Figure S7. VT $^1H\{^{31}P\}$ NMR spectrum of octaphosphane **2** in THF-d_8. The asterisks mark the decoalescing and emerging signals.

NMR Spectra of [AuCl(2)] (3)

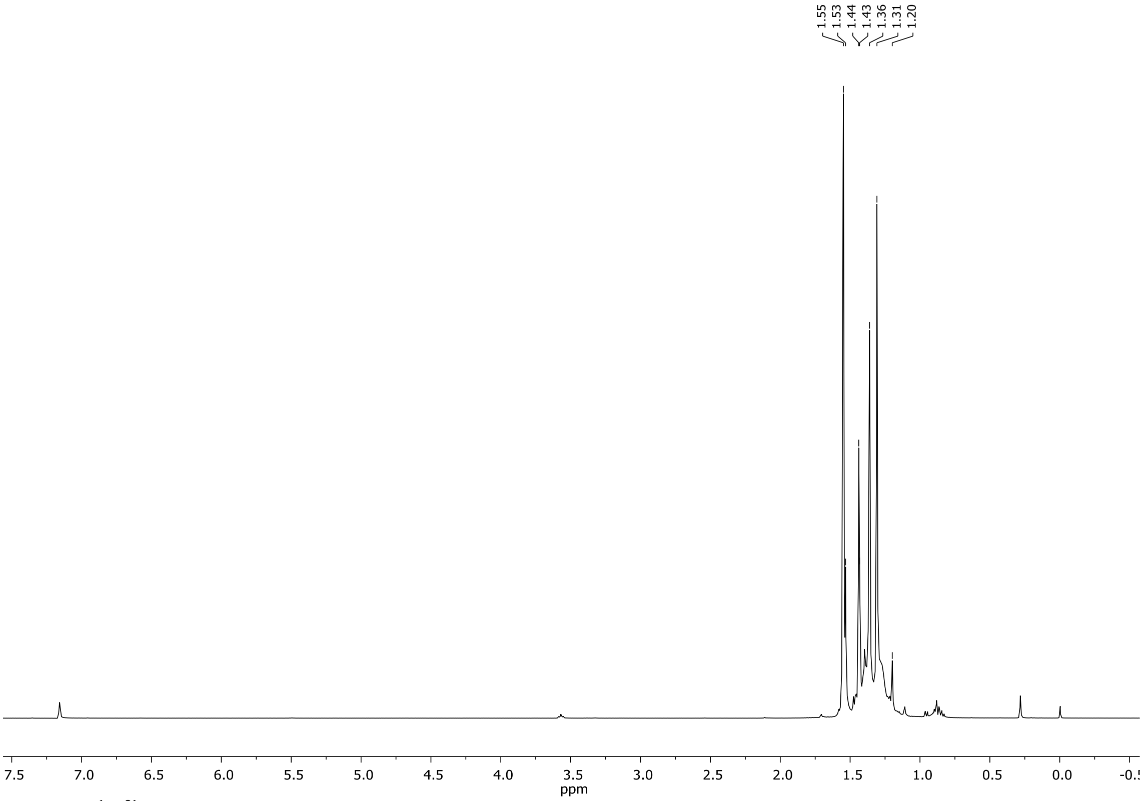

Figure S8. $^{1}H\{^{31}P\}$ NMR spectrum of complex **3** in THF-d_8.

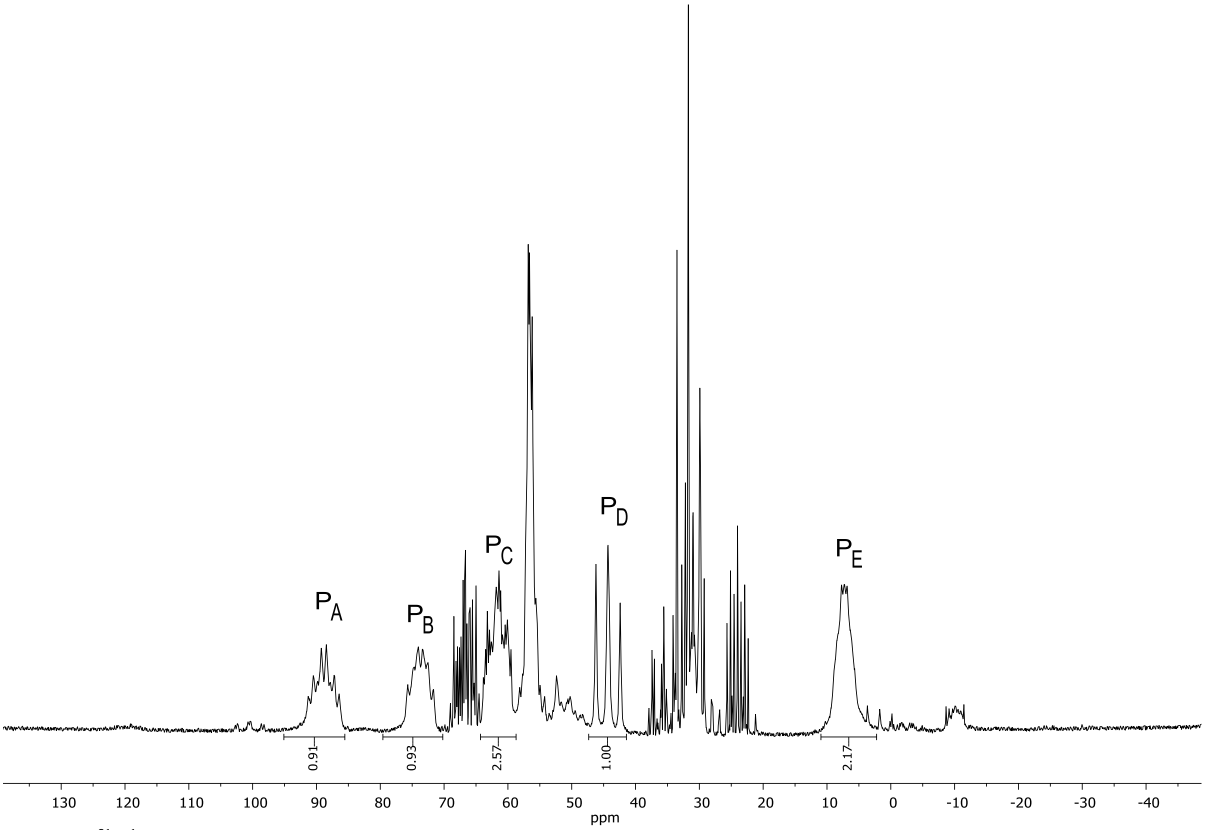

Figure S9. $^{31}P\{^{1}H\}$ NMR spectrum of complex **3** in THF-d_8. Additional signals for the octaphosphane **2** and the bimetallic complex **4** are detectable.

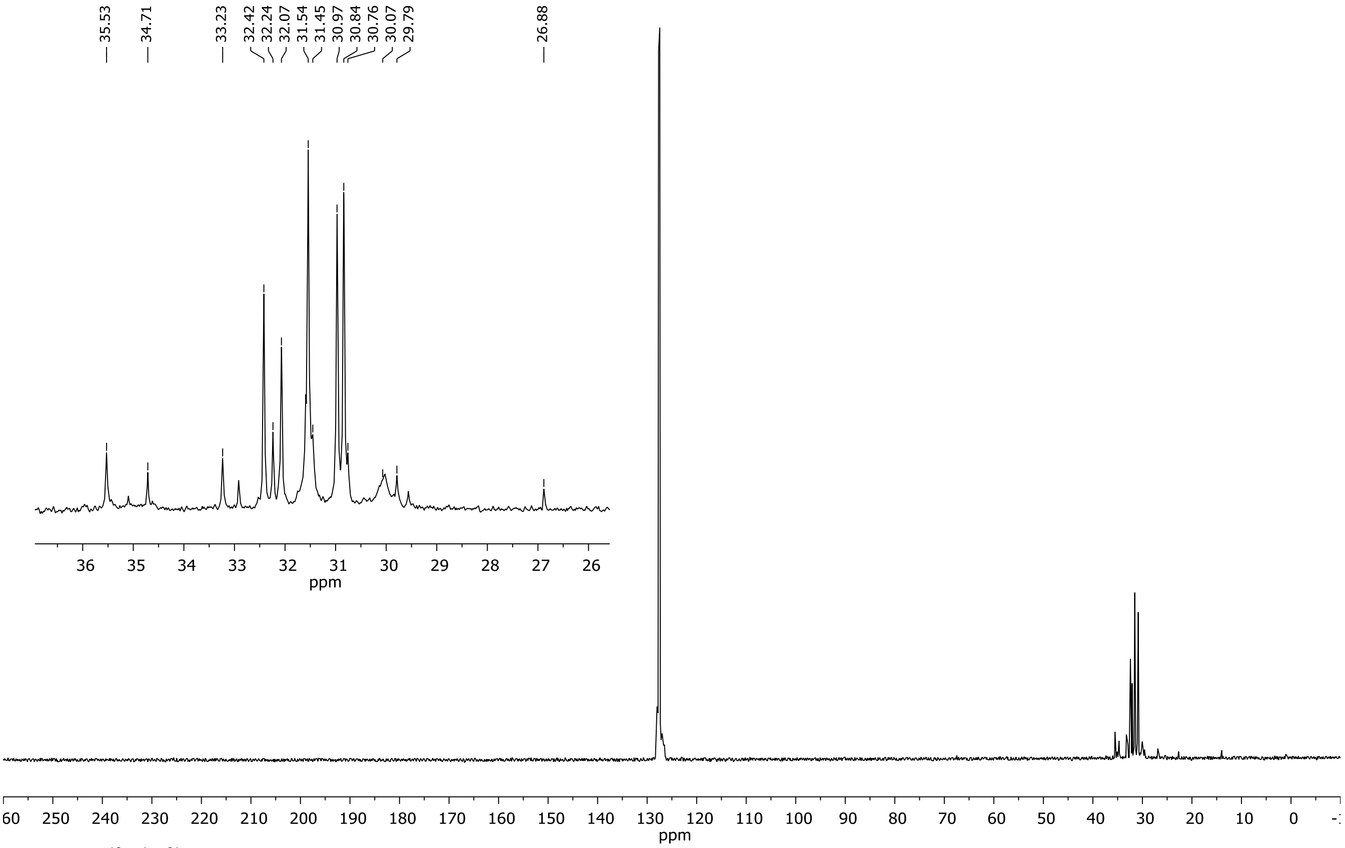

Figure S10. $^{13}C\{^{1}H,^{31}P\}$ NMR spectrum of complex **3** in THF-d_8.

NMR Spectra of [(AuCl)$_2$(2)] (4)

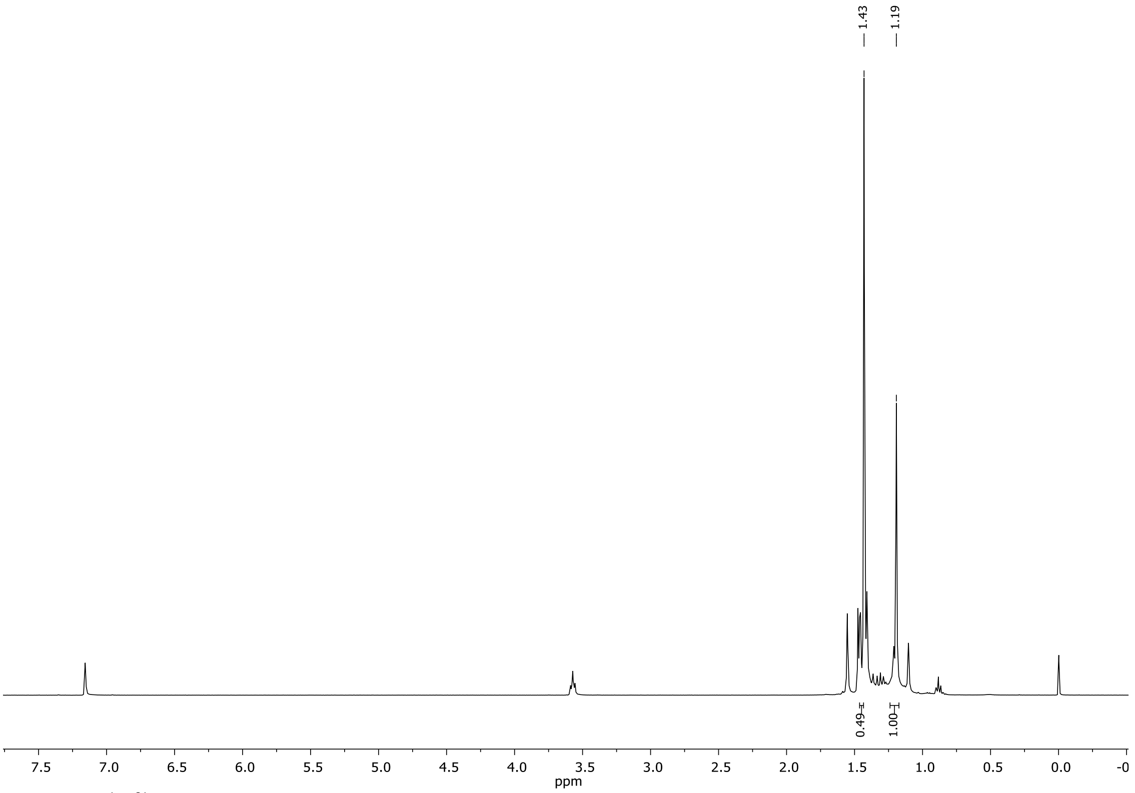

Figure S11. $^{1}H\{^{31}P\}$ NMR spectrum of complex **4** in THF-*d8*.

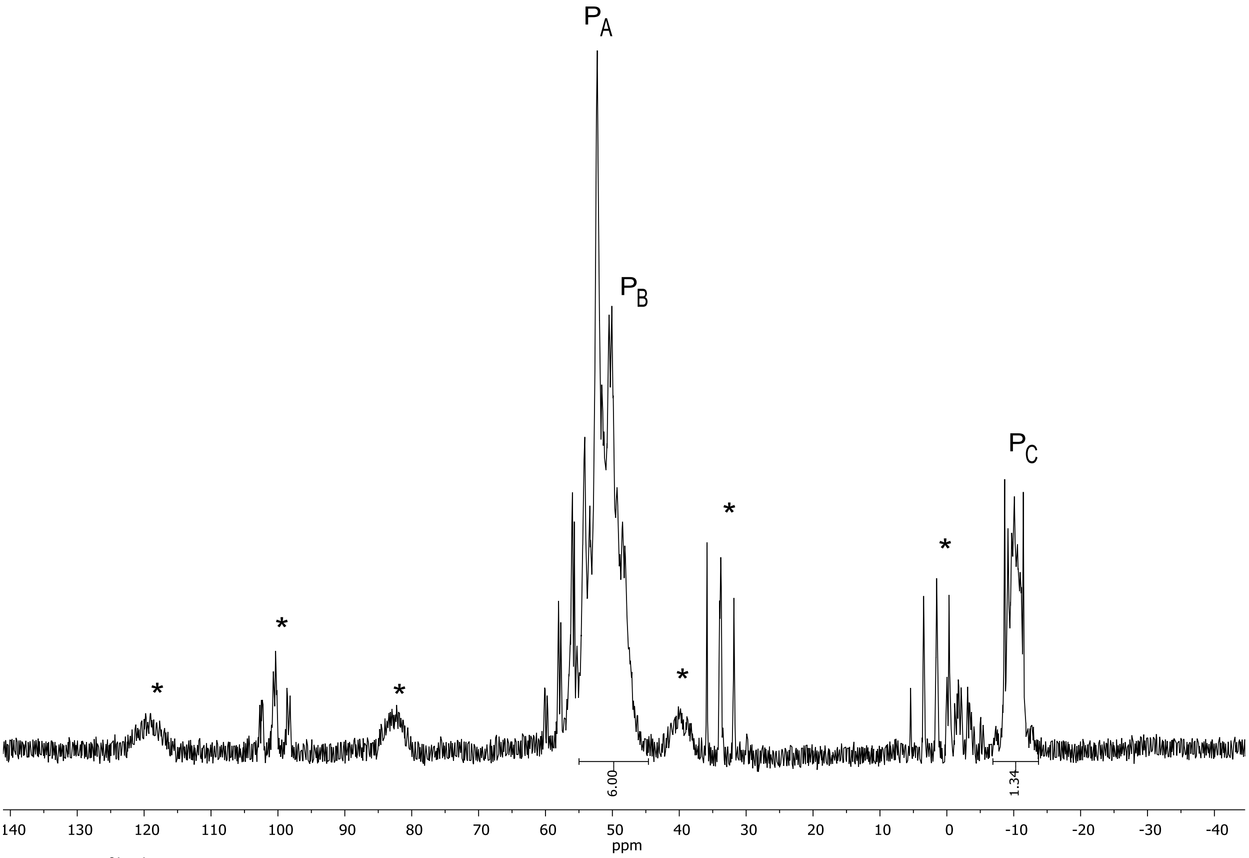

Figure S12. $^{31}P\{^{1}H\}$ NMR spectrum of complex **4** in THF-d_8. The asterisks mark signals, which correspond to the second constitutional isomer of the complex.

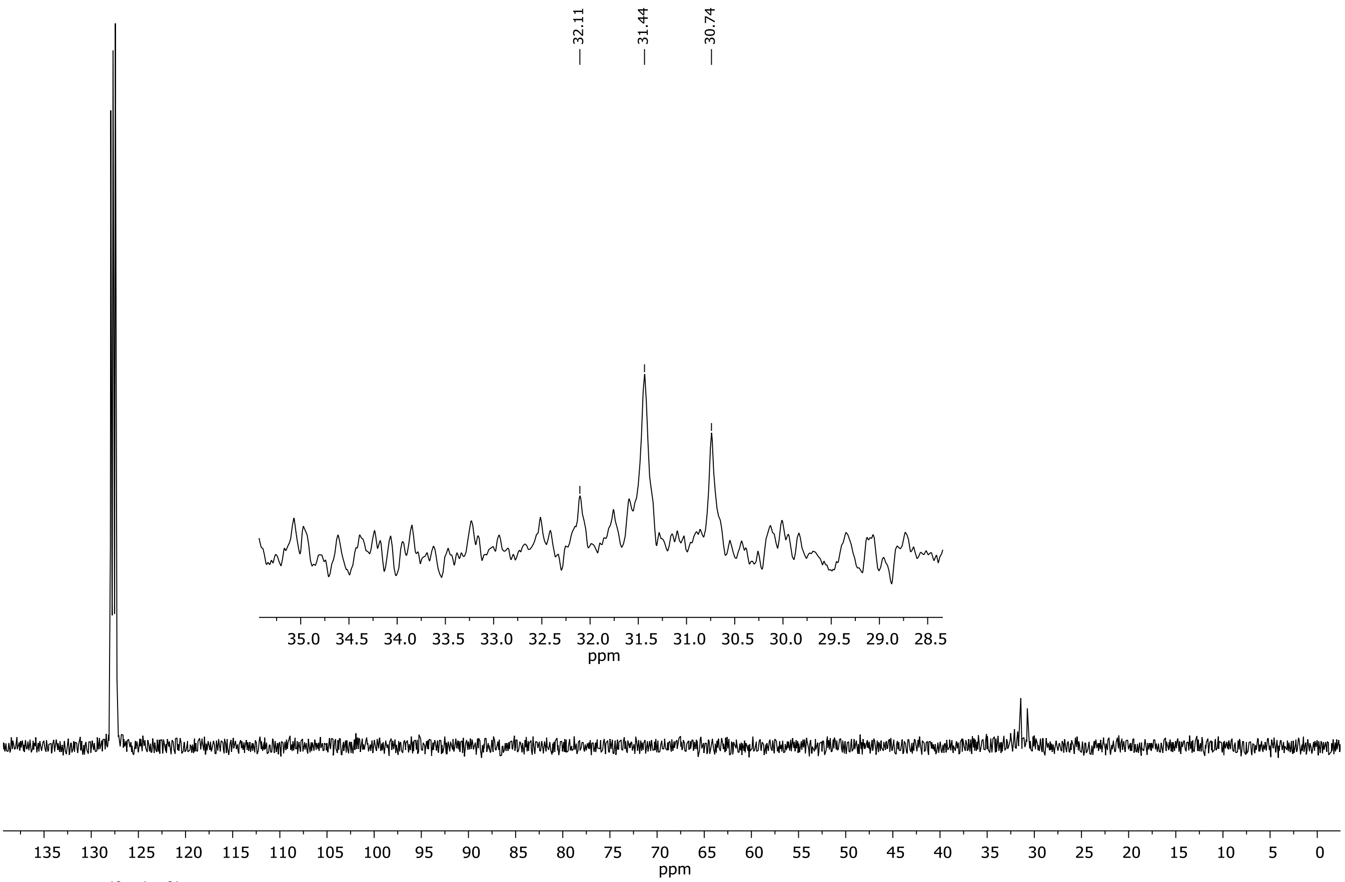

Figure S13. $^{13}C\{^{1}H,^{31}P\}$ NMR spectrum of complex **4** in THF-d_8.

NMR Spectra of [PdCl$_2$(2)] (5)

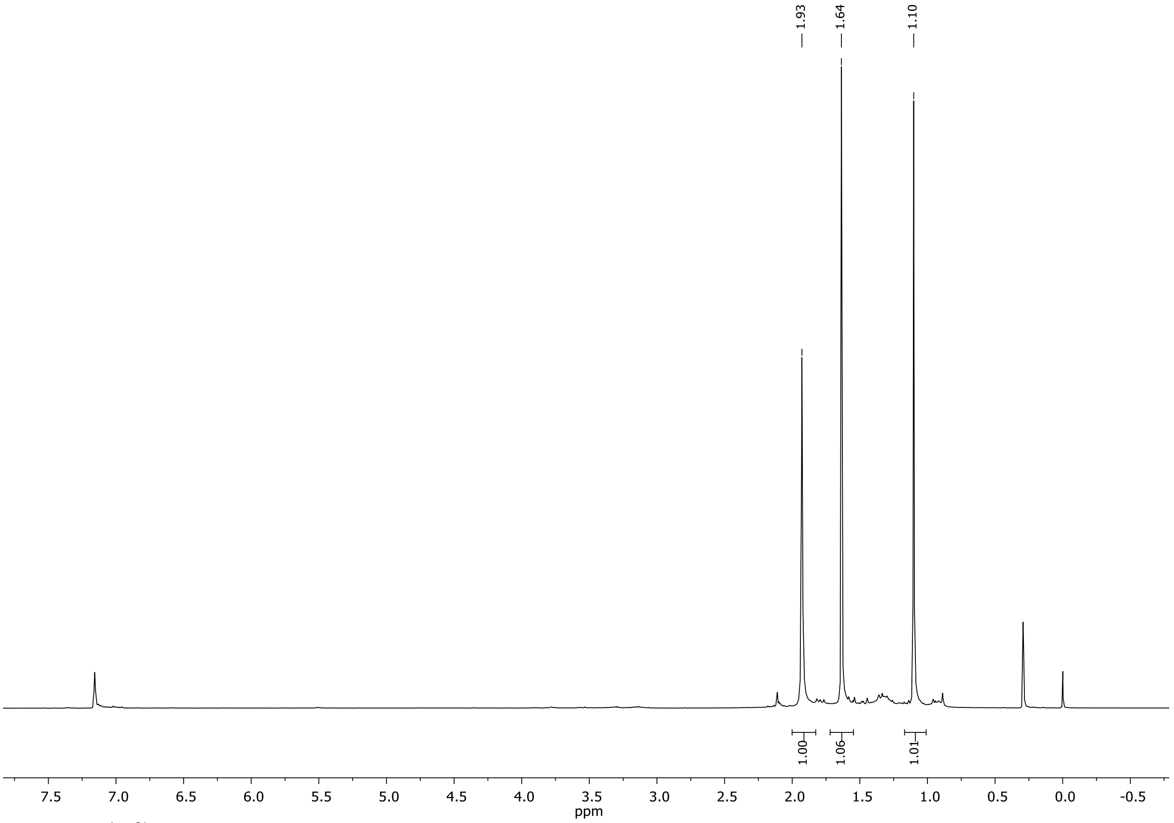

Figure S14. $^{1}H\{^{31}P\}$ NMR spectrum of complex **5** in C_6D_6.

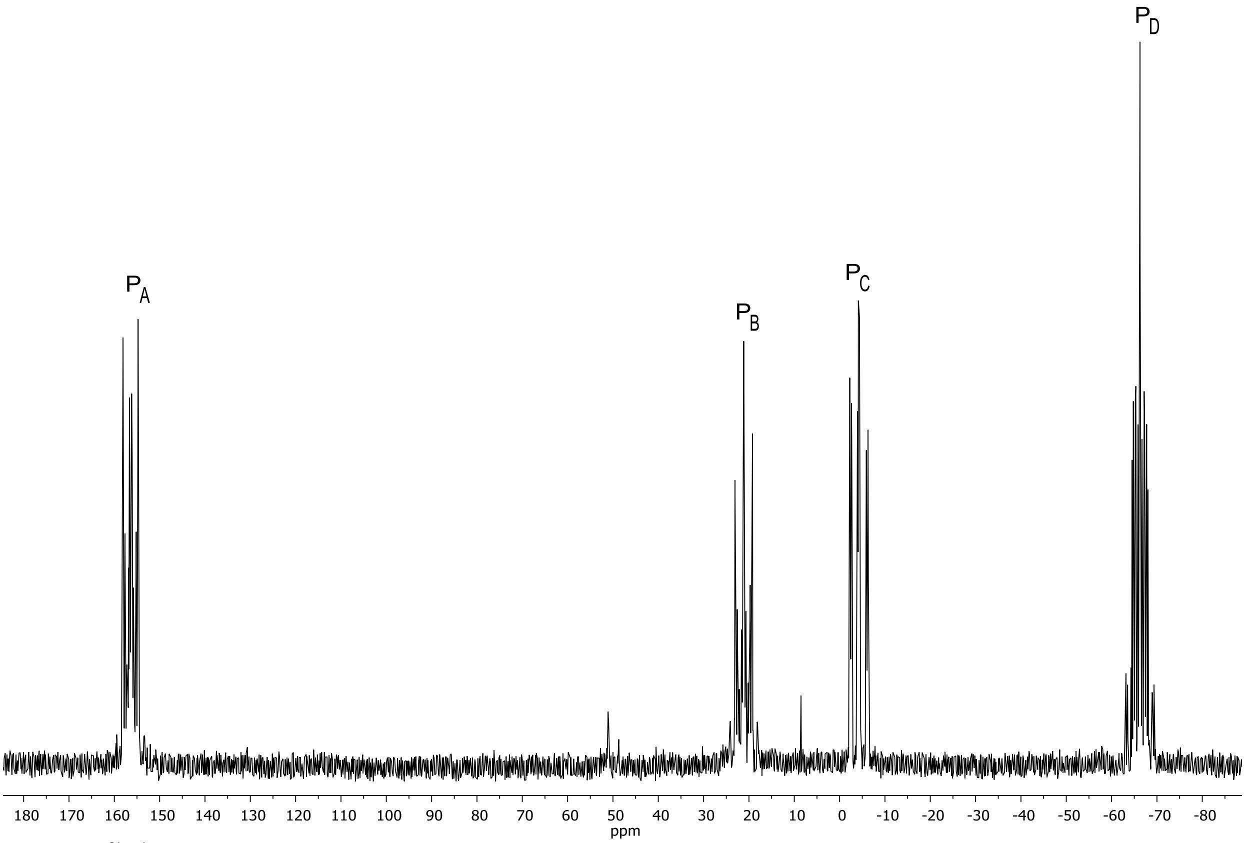

Figure S15. $^{31}P\{^{1}H\}$ NMR spectrum of complex **5** in C_6D_6.

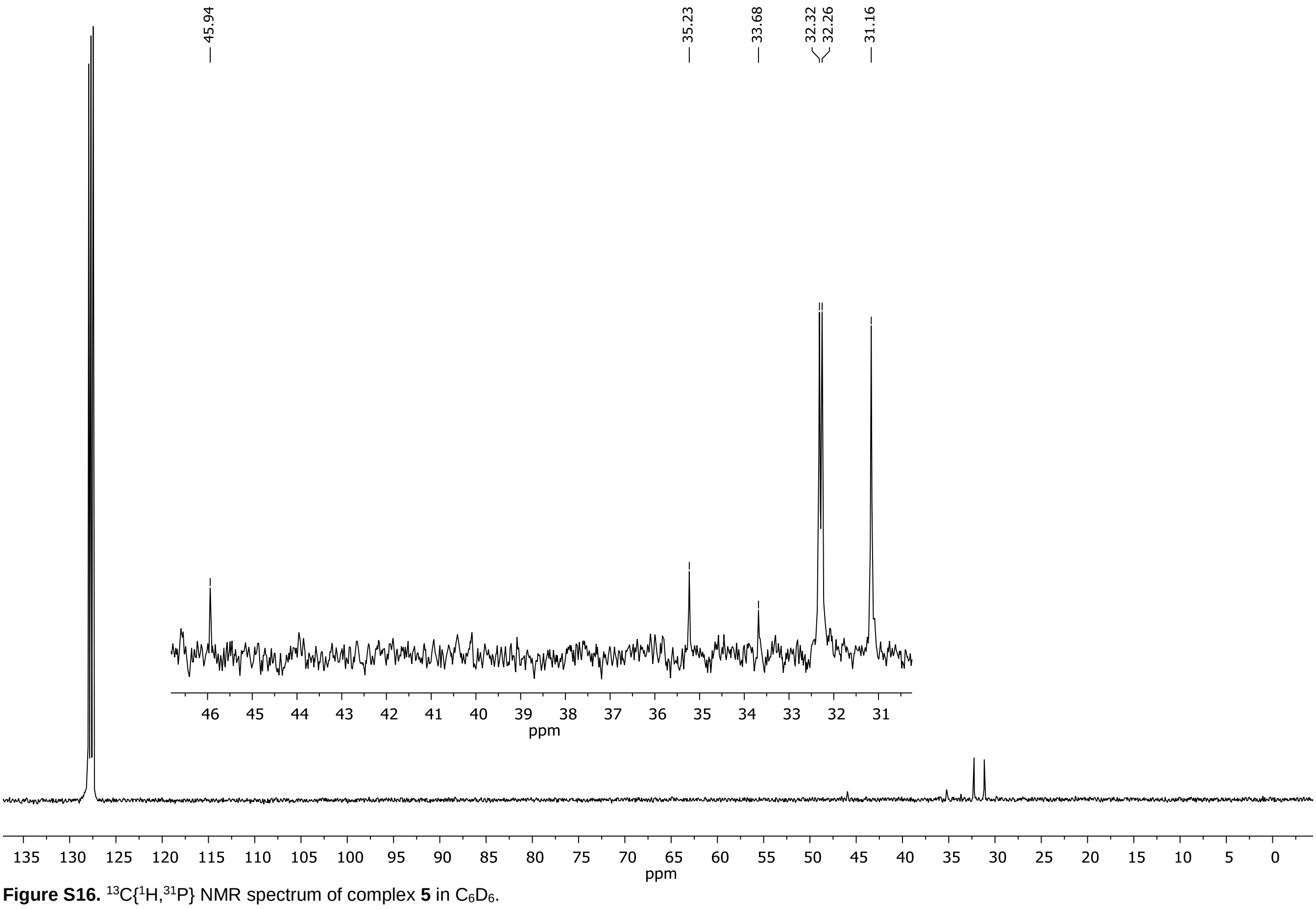

Figure S16. $^{13}C\{^{1}H,^{31}P\}$ NMR spectrum of complex **5** in C_6D_6.

4. Mass spectrometry measurements

Mass spectrometry measurements were carried out as ESI-MS with a BRUKER DALTONICS FT-ICR-MS spectrometer (Type APEX II, 7 Tesla).

The identity of **2** is supported by mass spectrometry. Two ions attributable to the monoxide of **2** are detected as adducts with H^+ or Na^+, respectively. They occur together with the higher oxides as a series of six peaks $[M(P_8{}^{t}Bu_6O_x)]^+$ (M = H, Na; x = 1–6) separated by *m/z* = 16. No peaks for $[M(P_8{}^{t}Bu_6O_x)]^+$ ions with x = 7 or 8 are detectable. This is in accordance with the observations from mass spectrometry of octaphosphane **1**[1] as well as the literature, which indicate that these octaphosphanes can be oxidized to the hexoxide but no further.[5] Obviously, the tendency of the P atoms that carry no *t*Bu group to be oxidized is extremely low. Another signal series that can be assigned to $[Na(P_8{}^{t}Bu_6)_2O_x]^+$ with x = 2–9 is found. No peak for the unoxidized octaphosphane is detectable. This could be due to lower ionization efficiency for the parent octaphosphane **2** or because it is swiftly oxidized under the conditions of the MS measurement.

5. X-Ray Diffraction

Single Crystal Measurements

Single crystal X-ray diffraction data were collected with a GEMINI CCD diffractometer (RIGAKU). The radiation source was a molybdenum anode (Mo-K_α, λ = 0.71073 Å). The absorption corrections were carried out semiempirically with the SCALE3 ABSPACK module[6]. All structures were solved by dual space methods with Sir-92[7] and SHELXT-2017. Structure refinement was done with SHELXL-2015/2018[8] by using full-matrix least-square routines against F^2. All hydrogen atoms were calculated on idealized positions. The pictures were generated with the program ORTEP3[9]. In all pictures, hydrogen atoms and solvent molecules were omitted for clarity and thermal ellipsoids are shown with 50% probability. CCDC 1956729 (**2**), CCDC 1956731 (**3**), CCDC 1956733 (**4**) and CCDC 1956734 (**5**) contain the supplementary crystallographic data for this paper. These data can be obtained free of charge via https://summary.ccdc.cam.ac.uk/structure-summary-form (or from the Cambridge Crystallographic Data Centre, 12 Union Road, Cambridge CB2 1EZ, UK; fax: (+44)1223-336-033; or deposit@ccdc.cam.ac.uk). Refinement special details are

- For the crystal structure of **2**: The crystal was non-merohedrally twinned, and the diffraction pattern was indexed based on two orientation matrices related by the twin law {0.177 0 –0.823 / 0 –1 0 / –1.177 0 –0.177} which can be interpreted as a twofold rotation about [3 0 –2]. The twin scale factor was refined to 0.21367. Both domains were integrated simultaneously and used for refinement.
- For the crystal structure of **3**: The complex consists of one octaphosphane molecule coordinated to an AuCl fragment close to a THF molecule. The THF molecule is partially replaced by a second AuCl fragment, which thus corresponds to the bimetallic complex **4**. The THF molecule and the Cl atom of the second fragment could only be refined isotropically. The occupancy was refined to 4.442%.
- For the crystal structure of **4**: There are contacts between the CH_3 (H11) group in the complex and the CH_2 (H25A) group of the solvent molecule. This is most likely no attractive intermolecular interaction but results from close packing of the molecules.

The dihedral angle λ in all molecular structures was extracted from the crystal structures as the mean value of the two dihedral angles between the two bridgehead phosphorus atoms and two phosphorus atoms which are connected to them and part of the same ring.

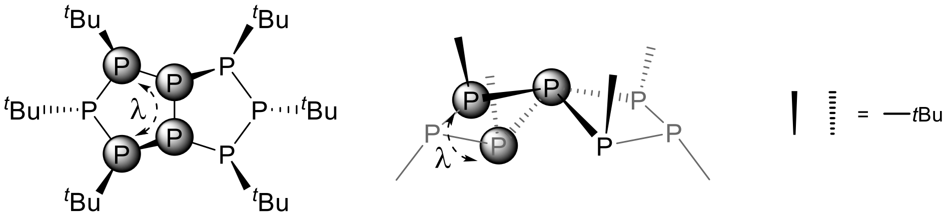

2

Figure S17. Illustration of the determination of the dihedral angle λ. The grey circles mark one set of atoms which were used for the determination.

SUPPORTING INFORMATION

Table S1. Summary of crystallographic data.

Molecule	$P_8{}^tBu_6$ (**2**)	[(AuCl)(**2**)]·THF (**3**·THF)	$[(AuCl)_2(\mathbf{2})]$·2THF (**4**·2THF)	$[PdCl_2(\mathbf{2})]\cdot C_6D_6)$ (**5**·C_6D_6)
**CCDC Number**	1956729	1956731	1956733	1956734
**Empirical formula**	$C_{24}H_{54}P_8$	$C_{31.82}H_{69.64}Au_{1.04}Cl_{1.04}O_{1.96}P_8$	$C_{32}H_{70}Au_2Cl_2O_2P_8$	$C_{30}H_{60}Cl_2P_8Pd$
**M_W (g·mol^{-1})**	590.43	974.18	1199.47	845.84
**Temperature (K)**	130(2)	130(2)	130(2)	130(2)
**Wavelength (Å)**	0.71073	0.71073	0.71073	0.71073
**Crystal system**	Monoclinic	Monoclinic	Monoclinic	Triclinic
**Space group**	$P2_1/n$	$P2_1/c$	$P2_1/c$	$P\overline{1}$
**a (Å)**	18.0664(6)	12.8286(2)	19.3577(7)	10.3154(3)
**b (Å)**	9.6931(3)	24.7998(4)	12.9700(3)	13.2668(3)
**c (Å)**	20.2852(8)	13.8358(3)	18.1816(6)	15.0083(4)
**α (°)**	90	90	90	76.450(2)
**β (°)**	110.560(4)	98.144(2)	92.153(3)	87.440(2)
**γ (°)**	90	90	90	85.901(2)
**Volume (Å^3)**	3326.1(2)	4357.4(1)	4561.6(2)	1990.82(9)
**Z**	4	4	4	2
**ρ (calc. in Mg·m^{-3})**	1.179	1.485	1.747	1.411
**μ (mm^{-3})**	0.432	3.909	6.850	0.942
**F(000)**	1272	1986	2360	880
**Crystal size (mm^3)**	0.15 x 0.10 x 0.07	0.40 x 0.30 x 0.20	0.08 x 0.07 x 0.05	0.15 x 0.15 x 0.07
**θ range for data collection (°)**	2.145 – 30.508	2.477 – 30.508	2.178 – 32.659	2.341 – 32.683
**Index ranges**	$-25 \le h \le 25$,<br>$-13 \le k \le 13$,<br>$-28 \le l \le 28$	$-18 \le h \le 18$,<br>$-31 \le k \le 35$,<br>$-17 \le l \le 19$	$-29 \le h \le 28$,<br>$-18 \le k \le 19$,<br>$-27 \le l \le 24$	$-14 \le h \le 15$,<br>$-19 \le k \le 20$,<br>$-22 \le l \le 20$
**Reflections collected**	14710	40426	47883	38891
**Independent reflections**	14710 [R(int) = 0.0618]	13287 [R(int) = 0.0329]	15274 [R(int) = 0.0932]	13196 [R(int) = 0.0307]
**Completeness (%) to 25.242°**	100.00	99.90	100.00	99.9
**Max. and min. transmission**	1.00000 and 0.98600	1.00000 and 0.36317	1.00000 and 0.62742	1.00000 and 0.93386
**Data / restraints / parameters**	14710 / 0 / 308	13287 / 0 / 404	15274 / 0 / 418	13196 / 0 / 388
**GooF on F^2**	0.917	1.086	1.018	1.016
**Final R indices [I>2σ(I)]**	R1 = 0.0569,<br>wR2 = 0.1010	R1 = 0.0355,<br>wR2 = 0.0843	R1 = 0.0597,<br>wR2 = 0.1202	R1 = 0.0281,<br>wR2 = 0.0629
**R indices (all data)**	R1 = 0.1239,<br>wR2 = 0.1136	R1 = 0.0450,<br>wR2 = 0.0889	R1 = 0.1296,<br>wR2 = 0.1450	R1 = 0.0350,<br>wR2 = 0.0661
**Largest diff. peak and hole e·Å^{-3}**	1.035 and –0.576	1.817 and –2.387	2.875 and –3.164	0.502 and –0.846

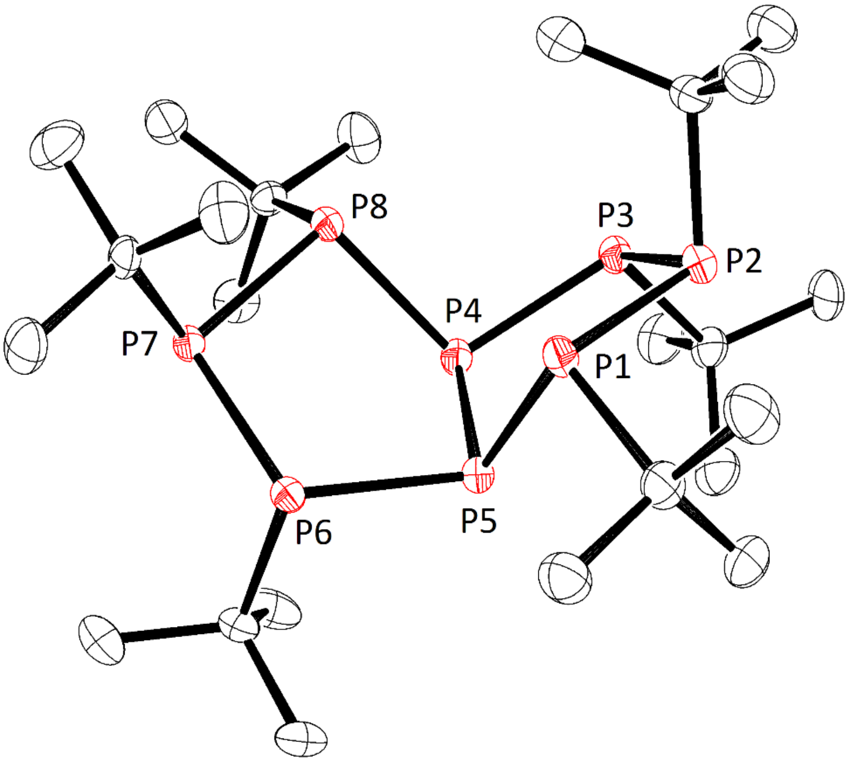

Figure S18. Molecular structure of octaphosphane **2**.

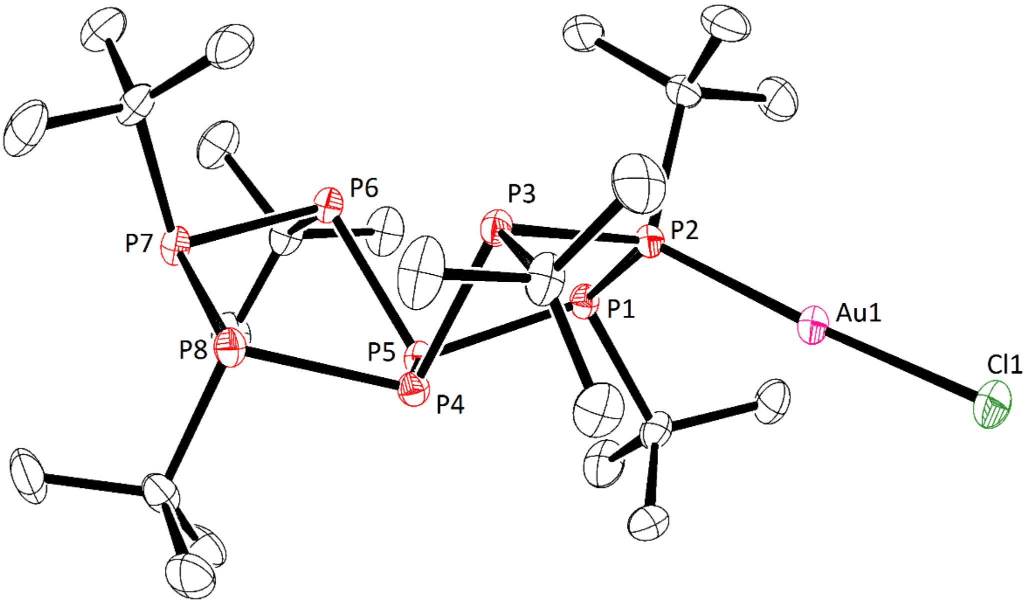

Figure S19. Molecular structure of monometallic complex **3**.

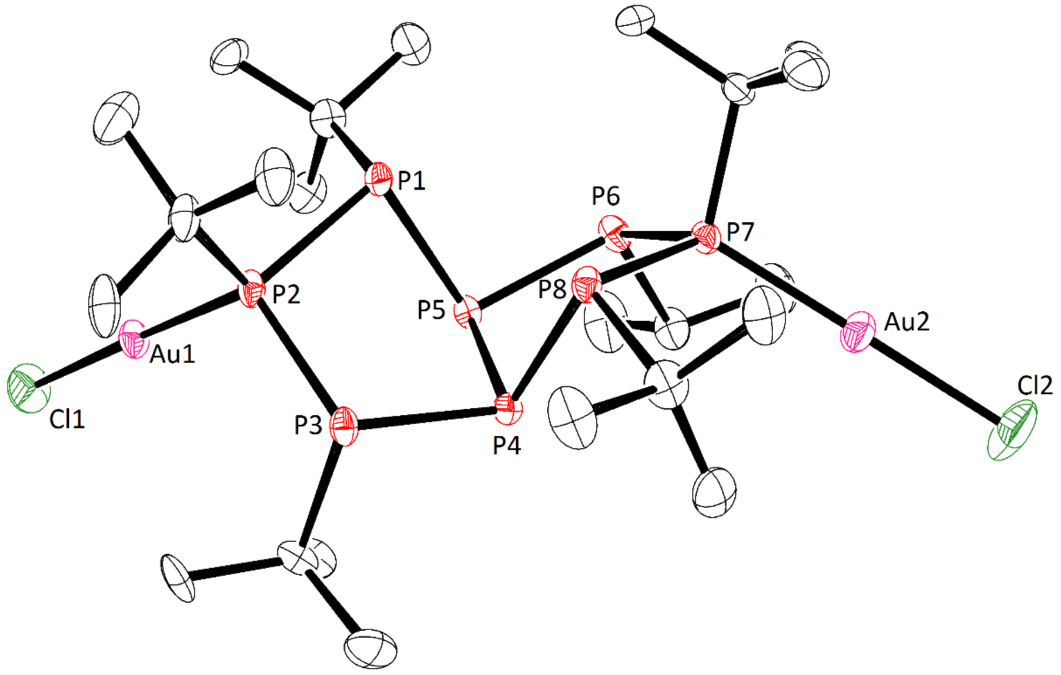

Figure S20. Molecular structure of homobimetallic complex **4**.

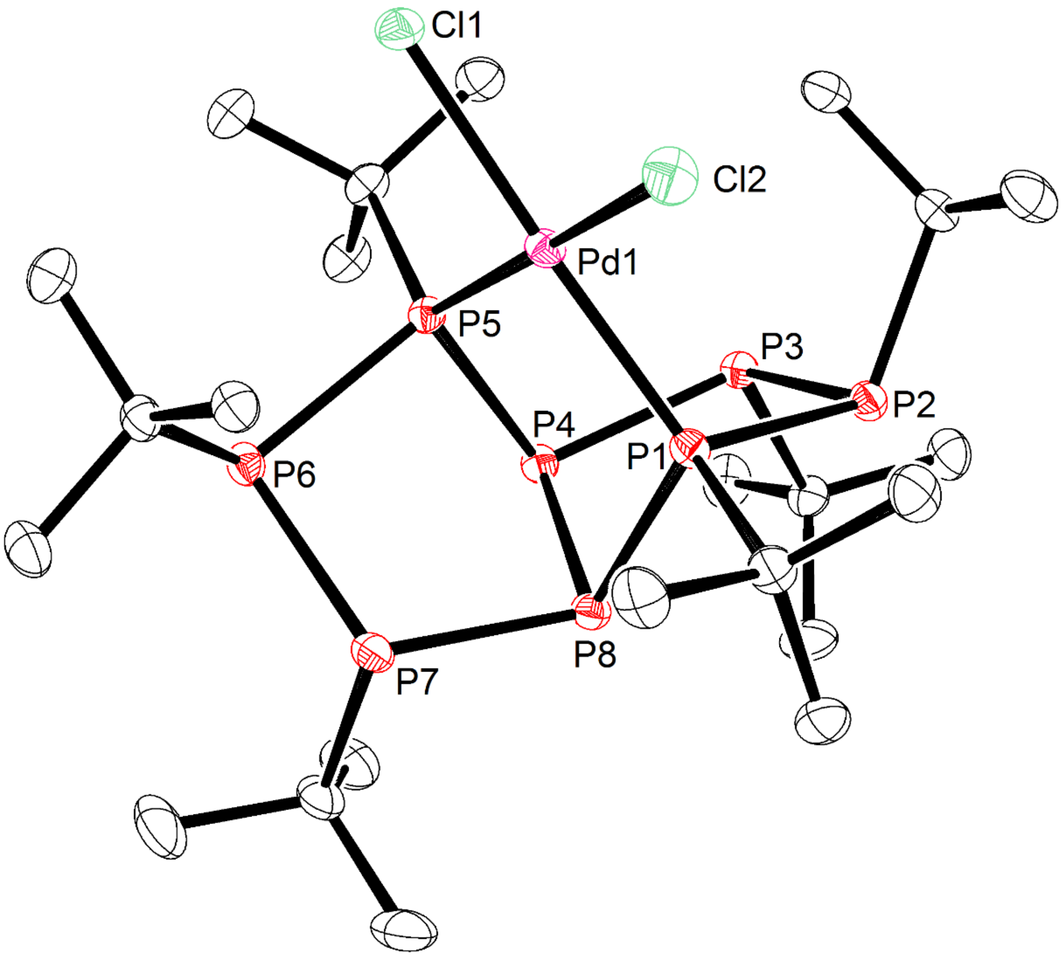

Figure S21. Molecular structure of complex **5**.

Powder X-ray Diffraction Measurements

Powder X-ray diffraction data were collected on a STADI-P diffractometer (STOE) with a silicon solid-state detector Methyn-1K (DECTRIS) at room temperature. The radiation source was a copper anode (Cu-K_α, λ = 1.540598 Å) combined with a germanium single crystal monochromator. Samples were measured in sealed glass capillaries (inner diameter 0.5 mm, HILGENBERG) with Debye-Scherrer geometry. Processing of the raw data was carried out with the diffractometer software WinXPow[10] (STOE). The theoretical powder pattern from the single crystal structures was calculated using MERCURY[11].

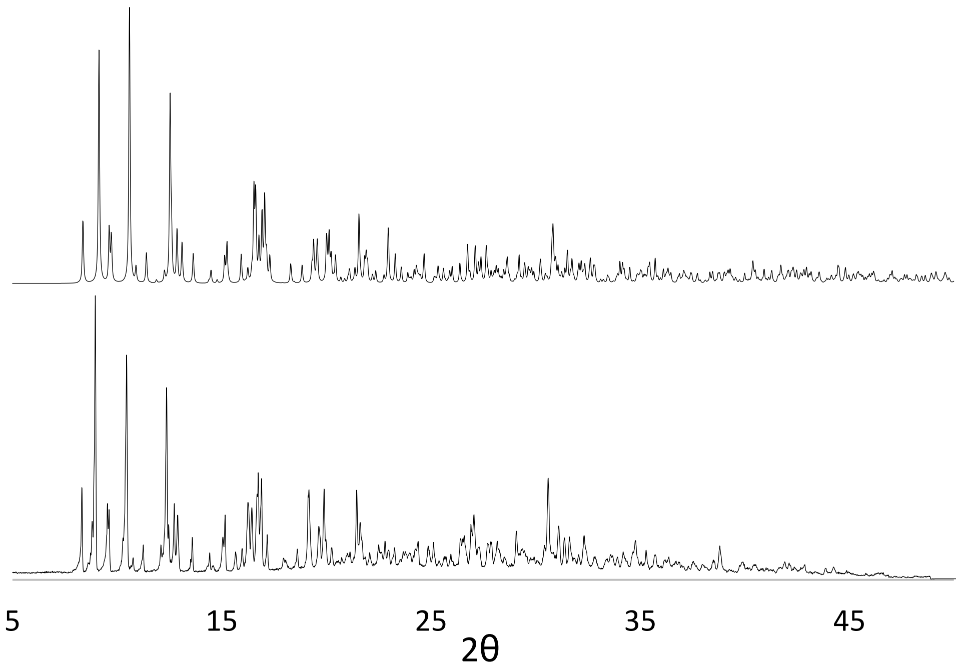

Figure S22. Calculated (top) and experimental (bottom) powder X-ray diffraction pattern of **4**.

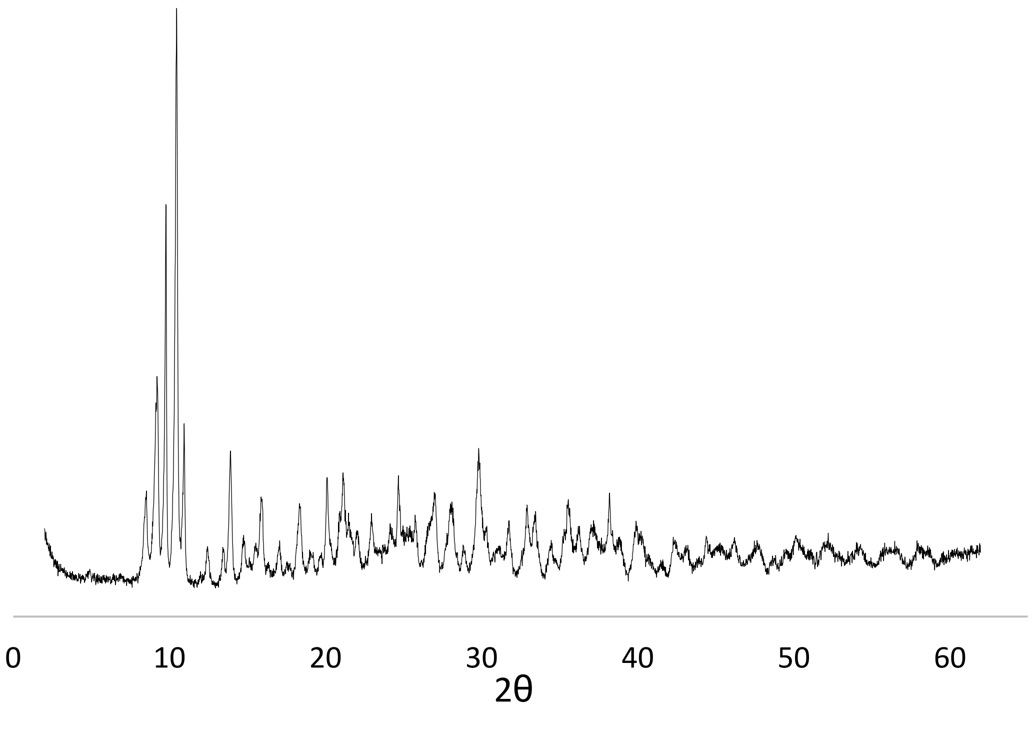

Figure S23. Powder X-ray diffraction pattern of **5**.

6. Quantum Chemical Calculations

General Remarks

All calculations were carried out with DFT using the program ORCA[12] (version 3.0.2). For all calculations, the convergence criterion was increased to 10^{-8} Hartree, the atom-pairwise dispersion correction D3[13] was used and, unless indicated otherwise, a simulation of the solvent environment was performed using the COSMO[14] model for which the dielectric constant was set to 7.58 (THF). Relativistic effects were considered using effective core potentials[15]. Furthermore, the RIJCOSX approximation was used to speed up the calculations.

Benchmark Study

The method for optimizing the molecular structures of the gold(I) complexes was benchmarked based on the molecular structure of the trinuclear complex of octaphosphane **1** obtained from single crystal XRD measurements and was also used for the gold complexes presented in this work.[1] The BP86 functional[16,17] in combination with the def2-SVP[18,19] basis set was used due to the best agreement with the experimental data.

Librational Profile of Octaphosphane 2

These calculations were carried out using the BP86[16,17] functional in combination with the def2-TZVP[18,19] basis set. A single point calculation of the optimized molecular geometries using the def2-TZVP basis set in combination with the PBE0[20,21] functional was performed to obtain the final energies. The transition state was located by a relaxed surface scan of the dihedral angle between the bridgehead P atoms and two of the directly connected P atoms, leaving the second one unconstrained. The transition state structures were verified as such by showing only one negative eigenvalue of the Hesse matrix, which corresponds to the described chemical motion.

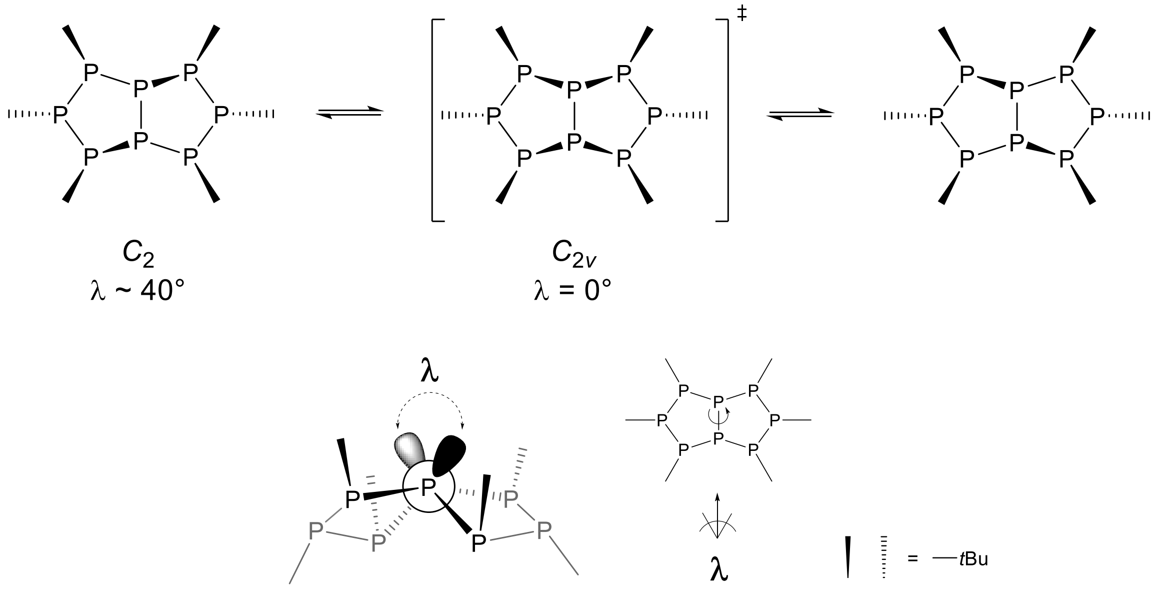

Scheme S1. Libration of octaphosphane **2**. The transition state corresponds to the average structure in solution. The remaining structures (left and right) correspond to the enantiomeric structures found in the solid state.

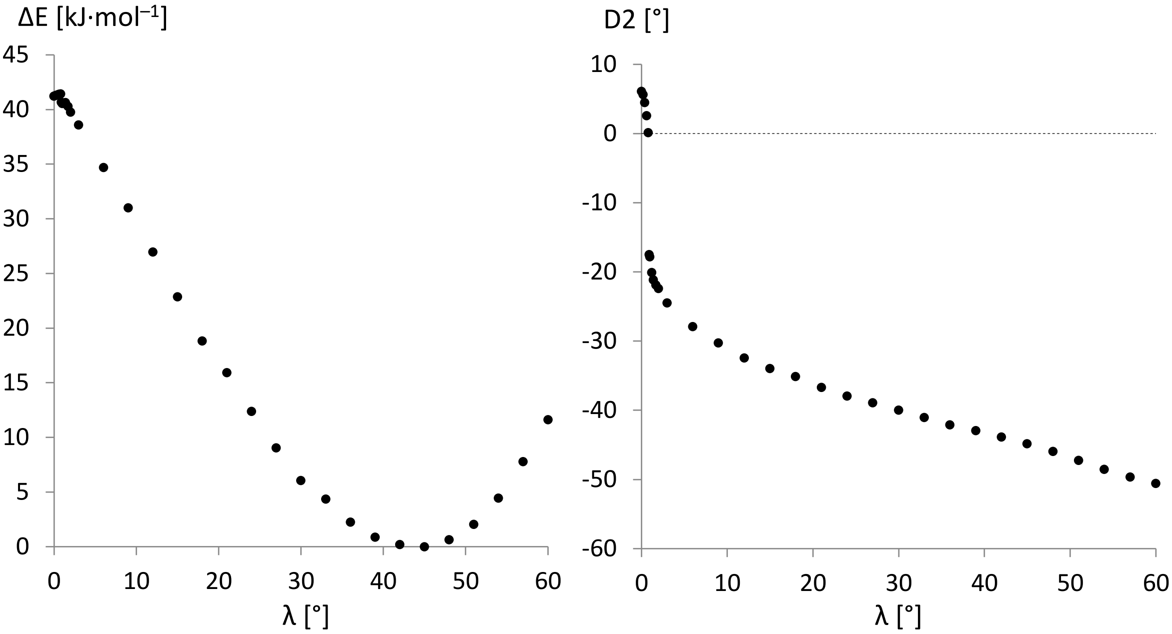

Figure S24. Librational profile of octaphosphane **2** with the relative energy (left) and the second, non-fixed dihedral angle (right) as a function of the fixed angle λ.

Mechanism of the Isomerization Reaction

These calculations were carried out using the B3LYP[22,23] functional in combination with the def2-TZVP[18,19] basis set.

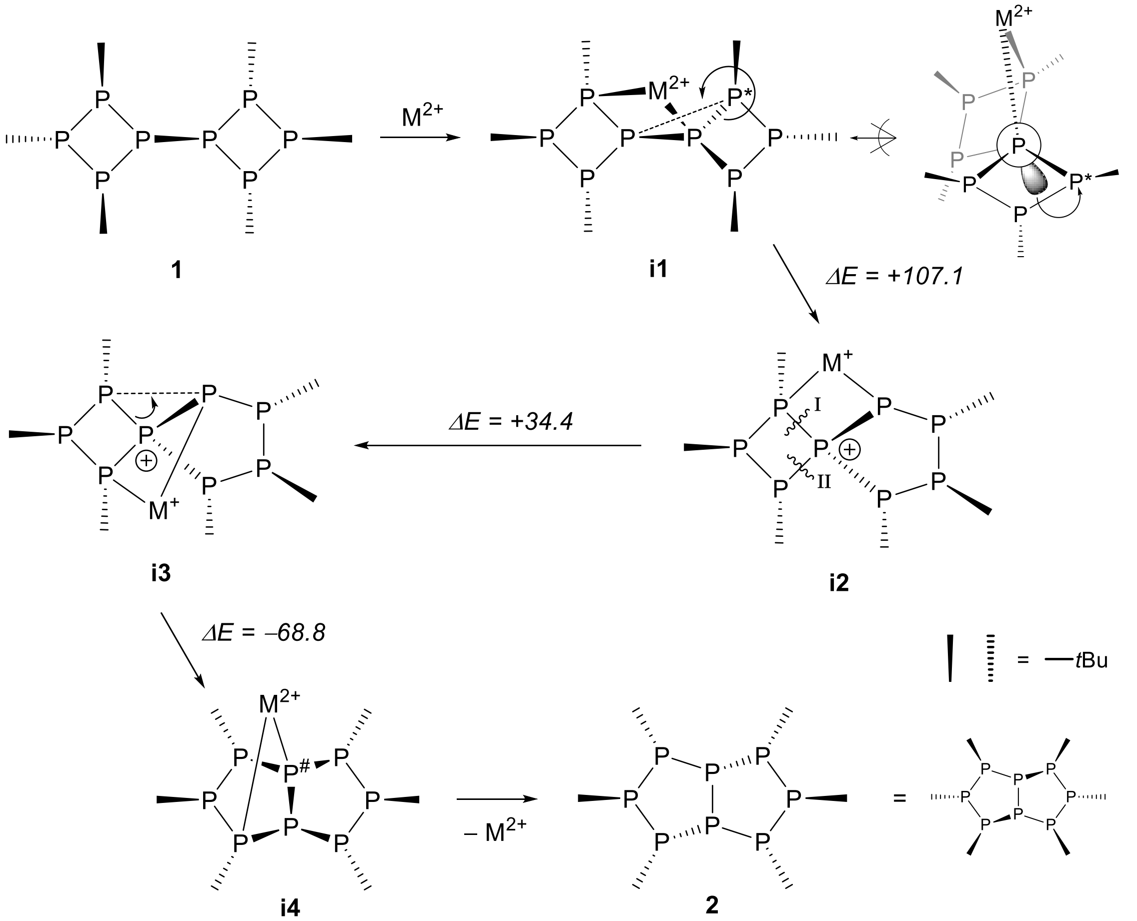

Scheme S2. Proposed mechanism of the isomerization reaction between octaphosphanes **1** and **2**. Reaction energies (calculated with Zn^{2+} as M^{2+} and two Cl^{-} coligands) are given in $kJ\cdot mol^{-1}$. Bond formation (dashed line) and cleavage (wavy lines) are indicated.

Comparison of Isomers of E_8R_6

The energies of different isomers were calculated with the combinations of functional and basis sets shown in Table S2. The relative energies of the different structures are displayed in Table S3.

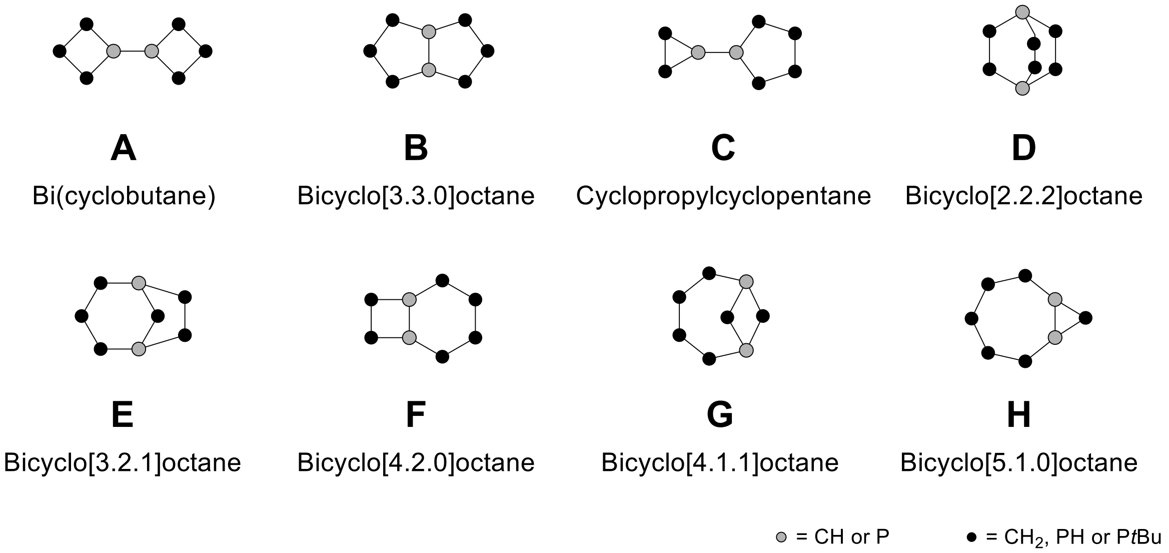

Scheme S3. Possible constitutional isomers of C_8H_{14}, P_8H_6 or P_8tBu_6. The names correspond to the organic molecules C_8H_{14}.

Table S2. Computational details for the energy calculation of the isomers of E_8R_6.

Isomer	Geometry optimization	Single point energy
C_8H_{14}	BP86[16,17]/def2-SVP[18,19]	PWPB95[24]/def2-TZVP
P_8H_6	B3LYP[22,23]/def2-TZVP[18,19]	PWPB95/def2-TZVP
$P_8{}^tBu_6$	B3LYP/def2-TZVP	PBE0[20,21]/def2-TZVPP[18,19]

Table S3. Relative energies ΔE in kJ·mol^{-1} for all isomers of C_8H_{14}, P_8H_6 or $P_8{}^tBu_6$. Letters correspond to constitutional isomers. The experimental values[25] ΔE(exp) for C_8H_{14} are referenced to the isomer **D**.

Structure	ΔE(exp)	ΔE(C_8H_{14})	ΔE(P_8H_6)	ΔE($P_8{}^tBu_6$)
**A**		172.7	101.4	28.2
**B**	13.3	12.1	0.0	0.0
**C**		104.7	70.0	92.8
**D**	7.4	7.4	45.4	41.1
**E**		0.0	36.4	130.6
**F**	81.1	97.3	101.1	60.4
**G**		88.1	94.5	93.9
**H**	89.8	103.1	78.9	180.2

7. References

[1] T. Grell, E. Hey-Hawkins, *Chem. Eur. J.* **2019**; submitted.
[2] G. R. Bourret, P. J. G. Goulet, R. B. Lennox, *Chem. Mater.* **2011**, *23*, 4954–4959.
[3] R. K. Harris, E. D. Becker, S. M. Cabral De Menezes, R. J. Goodfellow, P. Granger, *Concepts Magn. Reson.* **2002**, *14*, 326–346.
[4] J. P. Albrand, A. Cogne, J. B. Robert, *J. Am. Chem. Soc.* **1978**, *100*, 2600–2604.
[5] M. Baudler, J. Germeshausen, *Angew. Chem.* **1987**, *99*, 372–373; *Angew. Chem. Int. Ed. Engl.*, **1987**, *26*, 348–349.
[6] Agilent Technologies Ltd., *CrysAlis-Pro Software package*, **2014**.
[7] A. Altomare, G. Cascarano, C. Giacovazzo, A. Guagliardi, M. C. Burla, G. Polidori, M. Camalli, *J. Appl. Crystallogr.* **1994**, *27*, 435–436.
[8] G. M. Sheldrick, *Acta Cryst. A* **2015**, *71*, 3–8.
[9] L. J. Farrugia, *J. Appl. Crystallogr.* **2012**, *45*, 849–854.
[10] STOE Cie GmbH, *WinXPow*, **2014**.
[11] C. F. Macrae, P. R. Edgington, P. McCabe, E. Pidcock, G. P. Shields, R. Taylor, M. Towler, J. van de Streek, *J. Appl. Crystallogr.* **2006**, *39*, 453–457.
[12] F. Neese, *WIREs Comput. Mol. Sci.* **2012**, *2*, 73–78.
[13] a) S. Grimme, J. Antony, S. Ehrlich, H. Krieg, *J. Chem. Phys.* **2010**, *132*, 154104; b) S. Grimme, S. Ehrlich, L. Goerigk, *J. Comput. Chem.* **2011**, *32*, 1456–1465.
[14] S. Sinnecker, A. Rajendran, A. Klamt, M. Diedenhofen, F. Neese, *J. Phys. Chem. A* **2006**, *110*, 2235–2245.
[15] a) P. Schwerdtfeger, M. Dolg, W. H. E. Schwarz, G. A. Bowmaker, P. D. W. Boyd, *J. Chem. Phys.* **1989**, *91*, 1762–1774; b) F. Weigend, R. Ahlrichs, *Phys. Chem. Chem. Phys.* **2005**, *7*, 3297–3305.
[16] A. D. Becke, *Phys. Rev. A* **1988**, *38*, 3098–3100.
[17] J. P. Perdew, *Phys. Rev. B* **1986**, *33*, 8822–8824.
[18] A. Schäfer, H. Horn, R. Ahlrichs, *J. Chem. Phys.* **1992**, *97*, 2571–2577.
[19] A. Schäfer, C. Huber, R. Ahlrichs, *J. Chem. Phys.* **1994**, *100*, 5829–5835.
[20] J. P. Perdew, M. Ernzerhof, K. Burke, *J. Chem. Phys.* **1996**, *105*, 9982–9985.
[21] C. Adamo, V. Barone, *J. Chem. Phys.* **1999**, *110*, 6158–6170.
[22] K. Kim, K. D. Jordan, *J. Phys. Chem.* **1994**, *98*, 10089–10094.
[23] P. J. Stephens, F. J. Devlin, C. F. Chabalowski, M. J. Frisch, *J. Phys. Chem.* **1994**, *98*, 11623–11627.
[24] L. Goerigk, S. Grimme, *J. Chem. Theo. Comput.* **2011**, *7*, 291–309.
[25] A. Osmont, L. Catoire, I. Gökalp, *Energy Fuels* **2008**, *22*, 2241–2257.
